# Influence of quantity of additional food in achieving biological conservation and pest management in minimum-time for prey-predator systems involving Holling type III response

**DOI:** 10.1016/j.heliyon.2021.e07699

**Published:** 2021-08-03

**Authors:** Ananth V. S., Vamsi D. K. K.

**Affiliations:** Department of Mathematics and Computer Science, Sri Sathya Sai Institute of Higher Learning, Prasanthi Nilayam, Puttaparthi, Anantapur District, 515134 Andhra Pradesh, India

**Keywords:** Prey-predator systems, Additional food supplements, Optimal control problem, Bang-bang controls, Pontryagin's maximum principle

## Abstract

Incorporating additional food supplements into the predators' diet complementary to the target prey has gained importance over the years due to its pertinence in achieving biological conservation and biological control. Studies by theoretical ecologists and mathematicians reveal that by providing appropriate quality and quantity of additional food to the predator, the system could be driven either towards co-existence of species (to an admissible interior equilibrium), thereby achieving conservation or towards elimination of either of species achieving bio-control eventually with time. However, one of the limitations of these studies is that the desired state is reached only as asymptotes which makes the outcomes of the studies not that practically viable. In this work, to overcome the limitation of asymptotes, we formulate and study a time optimal control problem for additional food provided system involving type III response using quantity of additional food as the control. The objective of the study is to reach the desired terminal state in minimum time. To that end, we first prove the existence of optimal solution using the *Filippov's existence theorem* and then establish the characteristics of the optimal control using the *Pontryagin's Maximum Principle*. Using the Hamiltonian minimization condition and the monotonicity property of the Hamiltonian with respect to the quantity parameter, we show that the optimal control strategy is of bang-bang type with a possibility of multiple switches in the trajectory in case of biological conservation and no switch in case of pest management. Since the additional food system exhibits contrasting behaviour with respect to quality additional food, we have considered multiple cases of quality as a part of this study and in each case, we fixed the quality parameter as constant. The theoretical results have been illustrated by performing numerical simulations for various cases relating to both biological conservation and pest management. The theoretical outcomes of this study are in line with ecological field observations.

## 1 Introduction

Study of ecosystems where the predator is provided with alternate sources of food in addition to the target prey has gained prominence over the years and has become one of the important areas of research for biologists, theoretical and experimental ecologists, mathematicians and statisticians Harwood et al. (2004); Liu et al. (2018); Redpath et al. (2001); Sabelis et al. (2006); Sahoo and Poria (2014); Sen et al. (2015); Soltaniyan et al. (2020); Van Baalen et al. (2001); Wade et al. (2008). This is because provision of additional food has proven to be very effective in conserving endangered species Harwood et al. (2004); Putman and Staines (2004); Redpath et al. (2001) as well as controlling invasive or harmful species Prasad and Prasad (2018); Sabelis et al. (2006); Wade et al. (2008); Winkler et al. (2005). In particular, additional feeding for the purpose of supplementation is known to reduce the predation pressure on the prey by diverting the predator from the target prey Redpath et al. (2001). However, if additional food is not provided with proper care and vigilance, it could lead to counterproductive outcomes Putman and Staines (2004). On the other hand, to achieve bio-control, natural enemies of common pests are mass reared and released into the eco-systems to control the prey. These natural enemies are provided with additional food supplements, which affect their survival, gustatory response, fitness, fecundity and longevity Landis et al. (2000); Urbaneja-Bernat et al. (2013); Winkler et al. (2005) and this in turn helps in controlling pests in the system. Some of the outcomes of the mathematical studies of additional food systems Das and Samanta, 2018a, Das and Samanta, 2018b, Das and Samanta, 2020; Liu et al. (2018); Mondal and Samanta, 2019, Mondal and Samanta, 2020; Prasad and Prasad (2019); Sahoo and Poria (2014); Sen et al. (2015); Srinivasu et al., 2007, Srinivasu et al., 2018; Vamsi et al. (2019) reveal that the provision of additional food to the predators affects the global dynamics of the system causing indirect interaction of species. These findings are in line with the experimental observations when additional food is provided Harwood et al. (2004); Wade et al. (2008); Toft (2005).

The underlying assumption while studying these additional food systems is that the predators are generalist in nature and this makes them consume both the additional food supplements as well as their target prey Harwood et al. (2004); Van Baalen et al. (2001). Also, one of the important aspects of the prey-predator systems that play an important role in the global dynamics is the foraging behaviour of the predator, mathematically defined as the Functional Response Holling, 1959a, Holling, 1959b; Kot (2001). Among various functional responses exhibited by species, one response that is attributed to achieving stability in the system even at low prey densities is the Holling type III functional response, or the sigmoidal response which is displayed by many organisms in nature Elkinton et al. (2004); Fernández-Arhex and Corley (2005); Jamshidnia et al. (2010); Morozov (2010); Murdoch et al. (1975); Redpath and Thirgood (1999). For example, in a six year long study conducted to understand the functional response of the generalist predators hen harriers on Scottish grouse moors Redpath and Thirgood (1999), it was found that the harriers displayed type III response in preying grouse chicks. Also, from the ecological studies Elkinton et al. (2004), it was observed that generalist small mammals displayed type III response towards the alternative food sunflower seeds and type II response towards target prey gypsy moth pupae. This study shows that when the predator displays type II response towards the prey, then predation is seen highest at lowest prey densities causing outbreaks in the system. This is in contrast with the behaviour of systems involving type III response where rate of predation depends on the prey density. This is because the type III response depends on the prey detectability nature of the predator Srinivasu et al. (2018).

Recently, in the work Srinivasu et al. (2018), the authors have modeled and studied the following additional food provided prey-predator system involving type III functional response

(1.1) $$\frac{dN}{dT}=rN(1-\frac{N}{k})-\frac{cN^{2}}{a^{2}+N^{2}+\frac{h_{2}}{h_{1}}\frac{e_{2}}{e_{1}}A^{2}}P,$$

(1.2) $$\frac{dP}{dT}=(\frac{\epsilon_{1}cN^{2}+\epsilon_{2}c\frac{e_{2}}{e_{1}}A^{2}}{a^{2}+N^{2}+\frac{h_{2}}{h_{1}}\frac{e_{2}}{e_{1}}A^{2}})P-mP,$$

where *N* denotes the prey density, *P* the predator density and *A* the biomass of the additional food which is provided to the predators. Similar to the mathematical works mentioned above, we assume that *P* represents generalist predators which can feed on different diet supplements other than their target prey. The biological meaning of the parameters of the system (1.1) - (1.2) is provided in the Table 1.

**Table 1:** This table provides the biological meaning for the parameters used in the system (1.1) - (1.2).

Parameters	Biological meaning	Units
*r* > 0	Intrinsic Growth rate of the prey	1/time
*k* > 0	Prey carrying Capacity (Maximum prey density that eco-system can accommodate)	Dimensionless
*m* ≥ 0	Per-capita mortality rate of predators in the absence of prey	1/time
*e*_1_ > 0	Search time of the predator per unit prey availability	time
*e*_2_ > 0	Search time of the predator per unit additional food	time
*h*_1_ > 0	Handling time of predator per prey item	time
*h*_2_ > 0	Handling time of predator per unit additional food	time
$c=\frac{1}{h_{1}}>0$	Maximum rate of predation	1/time
$a=\frac{1}{\sqrt{e_{1}h_{1}}}>0$	Half-Saturation rate of Predators	1/time
*ϵ*_1_,0 < *ϵ*_1_ < 1	Nutritional Value of the prey	kcal
*ϵ*_2_,0 < *ϵ*_2_ < 1	Nutritional Value of the additional food	kcal
$b=\epsilon_{1}c=\frac{\epsilon_{1}}{h_{1}}>0$	Maximum growth rate of predators due to consumption of prey	kcal/time
$\alpha =\frac{\epsilon_{1}/h_{1}}{\epsilon_{2}/h_{2}}>0$	Ratio of maximum growth rate of predators due to prey with additional food	Dimensionless
$\eta =\frac{e_{2}\epsilon_{2}}{e_{1}\epsilon_{1}}>0$	Relative nutritional values of both items perceptible to predators	Dimensionless

Throughout this study, we assume that the maximum growth rate of the predators due to prey (denoted by *b*) is more than the mortality rate of the predators (denoted by *m*), i.e., we assume that b>m. Also, we assume that the additional food is provided regularly and uniformly to the predators. From the model perspective, the additional food density *A* is not dynamic but a constant. Additional food supplements can be alternative prey items but are manually supplied and provided by the eco-managers and they do not belong to the eco-system. From the system (1.1) - (1.2), we see that in the absence of the prey (N=0), the predators exponentially decay $\left(\frac{dP}{dT}=-mP\right)$. Similarly, in the absence of the predators (P=0), only the prey exist in the eco-system and they follow a logistic growth model $\left(\frac{dN}{dT}=rN(1-\frac{N}{k})\right)$ depending on the carrying capacity *k*. Further, we see that in the absence of additional food A=0, we get a type III system with only prey and predator components, also called the initial system Srinivasu et al. (2018). We request the readers to refer to Section 1 of the Appendix for the detailed derivation of the type III response in the presence of additional food.

With $\eta =\frac{e_{2}\epsilon_{2}}{e_{1}\epsilon_{1}}$, $\alpha =\frac{h_{2}\epsilon_{1}}{h_{1}\epsilon_{2}}$ and $b=\epsilon_{1}c$, the system (1.1) - (1.2) becomes

(1.3) $$\frac{dN}{dT}=rN(1-\frac{N}{k})-\frac{cN^{2}}{a^{2}+N^{2}+\alpha \eta A^{2}}P,$$

(1.4) $$\frac{dP}{dT}=b(\frac{N^{2}+\eta A^{2}}{a^{2}+N^{2}+\alpha \eta A^{2}})P-mP.$$

The parameter *α* given by $\left((\frac{\epsilon_{1}}{h_{1}})/(\frac{\epsilon_{2}}{h_{2}})\right)$ is the ratio between the maximum growth rates of the predator when it consumes the prey and additional food respectively. The parameter *α* essentially denotes the predator's relative efficiency of converting the available food sources into its own biomass. We see that *α* is inversely proportional to the nutritional value of additional food and directly proportional to the handling time of the additional food. The parameter $\eta =\frac{e_{2}\epsilon_{2}}{e_{1}\epsilon_{1}}$ denotes the ratio of search time of predator per unit food item of additional food and prey relative to nutritional values of additional food and prey. Thus, the term $\eta \frac{A^{2}}{N}=A((\frac{e_{2}A}{e_{1}N})/(\frac{\epsilon_{1}}{\epsilon_{2}}))$ represents the quantity of additional food discernible to the predator with respect to the prey relative to the nutritional value of prey to the additional food. We see that if (η<1)(η>1), then the quantity of additional food that is perceptible to the predator is less than (more than) the quantity of prey that is perceptible to the predator.

Now, letting $\gamma =\frac{k}{a},\;\beta =\frac{b}{r},\;\delta =\frac{m}{r}$ and $\kappa =\eta (\frac{A^{2}}{a^{2}})$ and using the dimensionless variables $x=\frac{N}{a},\;y=\frac{cP}{ra}\;\text{and}\;t=rT$, the system (1.3) - (1.4) gets converted to the non-dimensionalised system

(1.5) $$\frac{dx}{dt}=x(1-\frac{x}{\gamma })-(\frac{x^{2}y}{1+\alpha \kappa +x^{2}})$$

(1.6) $$\frac{dy}{dt}=\beta (\frac{x^{2}+\kappa }{1+\alpha \kappa +x^{2}})y-\delta y$$

Since we have assumed that b>m, we get that β>δ. Since the carrying capacity is strictly positive k>0, we get that the dimensionless parameter $\gamma =\frac{k}{a}>0$. In this study, we consider the parameter $\kappa =\eta (\frac{A^{2}}{a^{2}})$ to represent the **quantity** of additional food provided and *α* to represent the **quality** of additional food provided.

The outcomes of the work Srinivasu et al. (2018) emphasize the importance of quality and quantity of additional food that is provided to the predator supporting the inferences from the studies Kozak et al., 1994, Kozak et al., 1995; Putman and Staines (2004) and Winkler et al. (2005). In the work Srinivasu et al. (2018), the desired states are reached as asymptotes making the outcomes not that practically viable. To overcome this, we propose to study the system (1.5) - (1.6) further in the direction of achieving controllability in minimum (finite) time. Also, from ecological studies it is observed that the quantity of additional food plays a crucial role in the dynamics of the system Benelli et al. (2017); Urbaneja-Bernat et al. (2015); Vandekerkhove and De Clercq (2010); Wade et al. (2008); Winkler et al. (2005). Since the nutritional value and conversion factor of a food item can be treated as fixed, the quantity of consumption plays a major role in determining the effect of provision of the food item. It is also practically more feasible and much simpler to vary the quantity of food items than varying its quality. Thus, this study using the quantity of additional food is very relevant and significant.

Motivated by the aforementioned studies, in this article, we investigate the role of quantity of additional food *κ* in the global dynamics of the additional food provided system (1.5) - (1.6) in achieving the desired state in minimum (finite) time. This is done as follows: we determine the admissible states that could be reached by varying the quantity in the range $\left[\kappa_{\min},\kappa_{\max}\right]$. With the objective of reaching the desired admissible state in minimum time, we formulate and study a time optimal control problem with quantity of additional food as control parameter. We use *Filippov's existence theorem* to prove the existence of optimal solution and *Pontryagin's Maximum Principle* to obtain the characteristics of the optimal solutions. Throughout the study, we keep the quality of additional food *α* fixed. Since the dynamics vary completely depending on providing high quality or low quality of additional food, we analyze the optimal strategies for each case of fixed quality of additional food. The outcomes of this work can benefit eco-managers in providing additional food to species depending on the objective of biological conservation or pest management. The study towards achieving controllability of the system with respect to quality of additional food keeping the quantity fixed has its own relevance and is under progress Ananth and Vamsi (submitted for publication).

The section-wise division of this article is as follows: In the next section, we discuss the relevance of quantity as a control parameter. In section 3, we investigate the role of quantity of additional food in the dynamics of the type III additional food provided system and determine the admissible equilibria. In section 4, we formulate and study the time optimal control problem with quantity as control parameter following which in section 5, we discuss the nature of optimal strategies and applications to pest management. Then, in section 6, we illustrate the theory using numerical simulations. Finally, we present the discussion and conclusions in section 7.

## 2 Relevance of quantity of additional food in controllability

Some of the results of ecological and entomological studies show the crucial role played by quantity of additional food in habitat management especially in rearing of natural enemies to achieve bio-control Beach et al. (2003); Benelli et al. (2017); Davis et al. (2005); Put et al. (2012); Urbaneja-Bernat et al. (2015); Wade et al. (2008); Winkler et al. (2005); Zhao et al. (2003). Let us consider the example of *Nesidiocoris tenuis* Reuter (Hemiptera: Miridae) which is a zoophytophagous omnivorous predator found in the Mediterranean basin Arnó et al. (2010); Gabarra et al. (2008); Urbaneja et al. (2012). This mirid predator is mass-reared and released to control the pest species of tomato crop *Tuta absoluta* (Meyrick) (Lepidoptera: Gelechiidae) like the white-flies (Hemiptera: Aleyrodidae) Calvo et al. (2009); González-Cabrera et al. (2011); Mollá et al. (2011). The eggs of *Ephestia kuehniella* Zeller (Lepidoptera: Pyralidae) are well known factitious food that are used for rearing *N. tenuis* Mollá et al. (2014) but are extremely expensive for a long term usage. Some experiments were conducted to determine some other alternate sources of nutrition for *N. tenuis* Urbaneja-Bernat et al., 2013, Urbaneja-Bernat et al., 2015. When an adult couple of *N. tenuis* was released on the tomato plants and tested with four different quantities of *E. kuehniella* eggs with 0.1 g, 0.05 g, 0.02 g and none per plant in the fourth, the offspring development in each case showed significant results. There were no nymphs (offsprings) in the absence and when 0.02 g of *E. kuehniella* eggs were sprayed. Also, there were significantly greater number of nymphs in the case when 0.1 g eggs were provided compared to the provision of 0.05 g Urbaneja-Bernat et al. (2015). Also, when sugars were provided in combination with the *E. kuehniella* at two concentrations of 1M and 0.5M, the *N. tenuis* species consumed less eggs (40% reduction) of *E. kuehniella* with 0.5M of sugars Urbaneja-Bernat et al. (2013). Further, provision of 0.5M sucrose also leads to more immature survival of *N. tenuis* compared to 1M sucrose.

In many other cases, alternate foods are provided to the parasitoids of pest wasps to achieve bio-control Beach et al. (2003); Davis et al. (2005); Wade et al. (2008). Studies also show that their life span, mating ability, fecundity and gustatory response depend on the artificial food that is provided Benelli et al. (2017); Wade et al. (2008); Winkler et al. (2005). In Ellers et al. (2011), the effect of various concentrations of sugars was experimented on two parasitoid wasp species, *Asobara tabida* and *Trichopria drosophilae*. Both the species showed increase in longevity as the concentration on sucrose increased from 0% to 80% whereas when 100% concentration sucrose was provided, the longevity of both species drastically reduced. This shows that sometimes highest concentration foods could be detrimental to species Benelli et al. (2017). In case of the parasitoid *Diadegma semiclausum* which hosts on *Plutella xylostella* (diamondback moth), the effect of nectar and honeydew sugars showed that its gustatory response was high for 1M concentration of glucose and raffinose and it dipped as concentration reduced Winkler et al. (2005).

It can be inferred from the above discussion based on several experimental studies that the quantity of additional food supplied to the predators could be different at different stages depending on the nature of ingestion, quality of item and the bio-control strategy of the specific eco-system. These results show how crucial it is to provide appropriate quantity of additional food to the predators.

## 3 Role of quantity on global dynamics of the additional food system

In this section, we summarize the global dynamics of the additional food system (1.5) - (1.6) and the investigate the relevance of quantity on the dynamics. Since our focus is on controllability aspects of the system, we discuss only those details that will enable the understanding of further sections of this article . Readers are requested to refer to Srinivasu et al. (2018) to get a comprehensive understanding of the global dynamics and the stability analysis of the system (1.5) - (1.6).

From the analysis provided in Srinivasu et al. (2018), we see that the dynamics of additional food system depends on the existence and stability of interior equilibrium of the system in the absence of additional food. From the discussion in previous sections, we see that by considering the system (1.1) - (1.2) without alternative prey (by taking A=0), we obtain the initial system. Taking A=0 in the system (1.1) - (1.2) corresponds to κ=0 in the additional food system (1.5) - (1.6), so that the system (1.1) - (1.2) is simplified as follows:

$$\frac{dx}{dt}=x(1-\frac{x}{\gamma })-(\frac{x^{2}y}{1+x^{2}})\frac{dy}{dt}=\beta (\frac{x^{2}}{1+x^{2}})y-\delta y$$

The initial system admits three equilibrium points: the trivial equilibrium (0,0), the axial equilibrium (γ,0) and the interior equilibrium given by

$$\overset{¯}{x}=\sqrt{\frac{\delta }{\beta -\delta }},\;\;\overset{¯}{y}=(\frac{1+x^{2}}{x})(1-\frac{x}{\gamma })$$

The existence, stability and occurrence of Hopf bifurcation for the interior equilibrium of the initial system is summarized in the Table 2. Using these conditions as cases, we will study the impact of provision of additional food to the predators in each case.

**Table 2:** The global stability of the interior equilibrium and the limit cycle of the initial system are established in the article Srinivasu et al. (2018), the proofs of which are given in the Appendix of Srinivasu et al. (2018).

**Behaviour of the interior equilibrium point of the initial system**		
Case	Conditions	Nature of the interior equilibrium

*C* − *I*	$\gamma \leq \sqrt{\frac{\delta }{\beta -\delta }}\;\text{and}\;\gamma \leq 3\sqrt{3}$	Does not exist
*C* − *II*	$\gamma \leq \sqrt{\frac{\delta }{\beta -\delta }}\;\text{and}\;\gamma >3\sqrt{3}$	Does not exist
*C* − *III*	$\gamma >\sqrt{\frac{\delta }{\beta -\delta }}\;\text{and}\;\gamma \leq 3\sqrt{3}$	Exists and is Globally Asymptotically stable
*C* − *IV*	$\gamma >\sqrt{\frac{\delta }{\beta -\delta }}\;\text{and}\;3\sqrt{3}<\gamma \leq \frac{2\delta \sqrt{\delta }}{\left(\sqrt{\beta -\delta })(2\delta -\beta \right)}$	Exists and is Globally Asymptotically stable
*C* − *V*	$\gamma >\sqrt{\frac{\delta }{\beta -\delta }}\;\text{and}\;\frac{2\delta \sqrt{\delta }}{\left(\sqrt{\beta -\delta })(2\delta -\beta \right)}<\gamma$	Exists and is unstable admits Globally Stable Limit Cycle

Similar to the initial system, the additional food system (1.5) - (1.6) also possesses three equilibria: the trivial equilibrium (0,0), the axial equilibrium (γ,0) both of which always exist for the system, and the third, the interior equilibrium denoted by $\left(x^{⁎},y^{⁎}\right)$ with

$$x^{⁎}=\sqrt{\frac{\kappa (\delta \alpha -\beta )+\delta }{\beta -\delta }},\;y^{⁎}=(\frac{1+\alpha \kappa +x^{2}}{x})(1-\frac{x}{\gamma })$$

For the interior equlibrium to exist, we must have $x^{⁎}<\gamma$ and for the prey component $x^{⁎}$ to be well defined, we must have $(\frac{\kappa (\delta \alpha -\beta )+\delta }{\beta -\delta })>0$. Comparing the prey component of the interior equilibrium of the initial system $\overset{¯}{x}$ with that of the additional food system $x^{⁎}$, we see that $x^{⁎}=\overset{¯}{x}$ whenever $\alpha =\frac{\beta }{\delta }$. Suppose $\alpha \neq \frac{\beta }{\delta }$, then whenever $\alpha <\frac{\beta }{\delta }(\alpha >\frac{\beta }{\delta })$, we get $x^{⁎}<\overset{¯}{x}(x^{⁎}>\overset{¯}{x})$. This implies that the equilibrium level of prey in the additional food system is either more or less than that of the initial system depending on the relation of the parameter *α* (which represents the quality of additional food) to the ratio of the maximum birth rate of predators in the absence of additional food to the starvation rate of predators ($\frac{\beta }{\delta }$). Using the expressions of $\overset{¯}{x}$ and $x^{⁎}$, we can also observe that if the initial system does not admit interior equilibrium, then additional food can never admit equilibrium for $\alpha >\frac{\beta }{\delta }$. On the other hand, if $\alpha <\frac{\beta }{\delta }$, then the additional food system admits interior equilibrium even if the initial system does not. Thus, the authors in Srinivasu et al. (2018) have characterized additional food to be of high quality if $\alpha <\frac{\beta }{\delta }$ and of low quality if $\alpha >\frac{\beta }{\delta }$. The term Quality reflects the ability of the predators to control the prey by consuming the additional food. From the expressions of α,β and *δ* we see that the additional food is of high (low) quality if the maximum growth rate of the predators due to consumption of additional food ($\frac{\epsilon_{2}}{h_{2}}$) is greater (less) than the natural death rate (*m*) of the predators.

The existence and stability of the equilibria of the additional food system depends on the values assumed by the parameters of the system and the position of $x^{⁎}$ relative to the position of *γ*, the nature of the prey isocline and the condition of the initial system considered. From the phase space studies and stability analysis of the additional food system as in Srinivasu et al. (2018), we see that the eventual state and stability of the system can be determined based on the values assumed by the two exogenous parameters *α* and *κ* with respect to the following curves given below under each case of initial system presented in Table 2:

Prey Elimination Curve (PEC),

βκ−δ(1+ακ)=0

Transcritical Bifurcation Curve (TBC),

$\beta (\gamma^{2}+\kappa )-\delta (1+\gamma^{2}+\alpha \kappa )=0$

Hopf-bifurcation Curve (HBC),

$-2{\left(\kappa (\delta \alpha -\beta )+\delta \right)}^{\frac{3}{2}}-\gamma \sqrt{\left(\beta -\delta \right)}(\kappa (\alpha (2\delta -\beta )-\beta )+2\delta -\beta )=0$.

These three curves are obtained from the local stability analysis of each of the equilibria. For example, the stability of the trivial equilibria (0,0) depends on the determinant of the jacobian matrix evaluated at (0,0) which turns out to be βκ−δ(1+ακ). Thus, if βκ−δ(1+ακ)>0 (βκ−δ(1+ακ)<0), the equilibrium (0,0) is unstable (saddle). Now, let us consider the curve βκ−δ(1+ακ)=0. Based on the values of the parameters *α* and *κ*, if βκ−δ(1+ακ)>0 (βκ−δ(1+ακ)<0), then we say that the system tends to reach prey elimination with the disappearance of interior equilibria (system remains in co-existence of species). In particular, when βκ−δ(1+ακ)>0, the interior equilibrium which was unstable vanishes and the trivial equilibrium (0,0) which was a saddle becomes unstable. The axial equilibrium (γ,0) changes its stability from stable to saddle only if Hopf bifurcation occurs. Otherwise, its stability remains unaffected. Thus, the prey elimination curve, though not a typical bifurcation curve, shows that once the parameters cross this curve, the solution trajectories eventually reach prey elimination.

**Figure 1:**
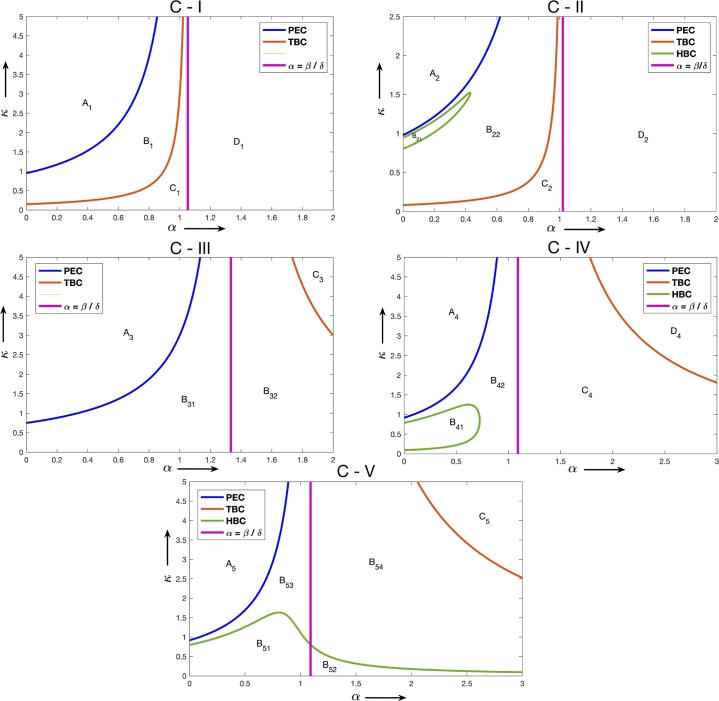
This figure depicts the division of the phase space by the prey elimination curve and two bifurcation curves: Hopf bifurcation curve and the transcritical bifurcation curve with respect to the parameters *α* and *κ*, for the additional food system (1.5) - (1.6) under Cases - C - I – C - V. The parameter values considered for each of the cases in the above plot are as follows: (i) Case C - I: *β* = 0.5, *δ* = 0.475, and *γ* = 4; (ii) Case C - II: *β* = 0.5, *δ* = 0.49, and *γ* = 6.7; (iii) Case C - III: *β* = 0.4, *δ* = 0.3, and *γ* = 3; (iv) Case C - IV: *β* = 0.24, *δ* = 0.22, and *γ* = 7; (v) Case C - V: *β* = 0.24, *δ* = 0.22, and *γ* = 8.

The transcritical bifurcation curve is obtained from the stability analysis of the axial equilibrium (γ,0) and the existence of interior equilibrium and Hopf bifurcation curve is obtained from the stability analysis of the interior equilibrium. Since the two parameters *α* and *κ* are crucial for the additional food system establishing the impact of additional food, the study of the state of the system using the above curves with respect to change in these two parameters gives us the global picture of the dynamics for the additional food system. Plotting these curves with respect to the parameters *α* and *κ* along with the curve $\alpha =\frac{\beta }{\delta }$, we get the diagrams shown in Fig. 1. From these diagrams, we see that the prey elimination curve and the transcritical bifurcation curve both are asymptotic to the curve $\alpha =\frac{\beta }{\delta }$ and under each case of the initial system provided in the Table 2, we see that the additional food system undergoes varied dynamics. Based on the diagrams in Fig. 1 and the detailed dynamics presented in Srinivasu et al. (2018), we summarize the global dynamics for the additional food system in the Table 3.

**Table 3:** Dynamics of additional food provided system.

Regions	Conditions		Nature of the equilibria		
	$\gamma \leq \sqrt{\frac{\delta }{\beta -\delta }}$	$\gamma \leq 3\sqrt{3}$	*E*_0_ = ((0,0))	*E*_1_ = ((*γ*,0))	*E*^⁎^ = ((*x*^⁎^,*y*^⁎^))
*D*_1_			saddle	stable	Does not Exist
*C*_1_			saddle	stable	Does not Exist
*B*_1_			saddle	unstable	stable
*A*_1_			unstable	saddle	Does not Exist
	$\gamma \leq \sqrt{\frac{\delta }{\beta -\delta }}$	$\gamma >3\sqrt{3}$	*E*_0_ = ((0,0))	*E*_1_ = ((*γ*,0))	*E*^⁎^ = ((*x*^⁎^,*y*^⁎^))
*D*_2_			saddle	stable	Does not Exist
*C*_2_			saddle	stable	Does not Exist
*B*_22_			saddle	saddle	stable
*B*_21_			saddle	saddle	unstable G.A.S. limit cycle
*A*_2_			unstable	saddle	Does not Exist
	$\gamma >\sqrt{\frac{\delta }{\beta -\delta }}$	$\gamma \leq 3\sqrt{3}$	*E*_0_ = ((0,0))	*E*_1_ = ((*γ*,0))	*E*^⁎^ = ((*x*^⁎^,*y*^⁎^))
*B*_31_			saddle	saddle	stable
*B*_32_			saddle	saddle	stable
*C*_3_			saddle	stable	Does not Exist
*A*_3_			unstable	saddle	Does not Exist
	$\gamma >\sqrt{\frac{\delta }{\beta -\delta }}$	$3\sqrt{3}<\gamma \leq \frac{2\delta \sqrt{\delta }}{\left(\sqrt{\beta -\delta })(2\delta -\beta \right)}$	*E*_0_ = ((0,0))	*E*_1_ = ((*γ*,0))	*E*^⁎^ = ((*x*^⁎^,*y*^⁎^))
*B*_43_			saddle	unstable	stable
*B*_42_			saddle	unstable	stable
*B*_41_			saddle	saddle	unstable G.A.S. limit cycle
*C*_4_			saddle	stable	Does not Exist
*A*_4_			unstable	saddle	Does not Exist
	$\gamma >\sqrt{\frac{\delta }{\beta -\delta }}$	$\frac{2\delta \sqrt{\delta }}{\left(\sqrt{\beta -\delta })(2\delta -\beta \right)}<\gamma$	*E*_0_ = ((0,0))	*E*_1_ = ((*γ*,0))	*E*^⁎^ = ((*x*^⁎^,*y*^⁎^))
*B*_51_			saddle	unstable	unstable G.A.S. limit cycle
*B*_52_			saddle	unstable	unstable G.A.S. limit cycle
*B*_53_			saddle	saddle	stable
*B*_54_			saddle	saddle	stable
*C*_5_			saddle	stable	Does not Exist
*A*_5_			unstable	saddle	Does not Exist

Since we wish to drive the system to a desired state in minimum time using quantity of additional food as the control parameter, in this section, we will view the dynamics of the system (1.5) - (1.6) from the perspective of quantity of additional food represented by the parameter *κ*. The Table 4 summarizes the dynamics of the additional food system with respect to *κ*. As discussed earlier, the state of the system and the stability of the equilibria can be determined by the values of *α* and *κ* with respect to the two bifurcation curves along with the prey elimination curve. Since this study is essentially about the role of quantity of additional food, hereafter, we shall assume the quality of additional food to be constant. Thus, we will study the dynamics of the system by varying the quantity of additional food supply for a fixed quality.

**Table 4:** Global dynamics of the system (1.5) - (1.6) w.r.t. the parameter *κ*.

Range of *α*	Condition	Nature of equilibria			Behaviour of trajectories	Applicable cases
		(0,0)	(*x*^⁎^(*κ*),*y*^⁎^(*κ*))	(*γ*,0)		
$0<\alpha <\frac{\beta }{\delta }$	*κ* < *P*	Saddle	Does not Exist	Stable	Towards (*γ*,0)	*C*_1_, *C*_2_
	*P* < *κ* < *R*	Saddle	Exists and Stable	Unstable	Towards (*x*^⁎^(*κ*),*y*^⁎^(*κ*))	*C*_1_, *C*_2_
	*P* < *κ* < *Q* < *R*	Saddle	Exists and Stable	Unstable	Towards (*x*^⁎^(*κ*),*y*^⁎^(*κ*))	*C*_2_
	0 < *κ* < *Q* < *R*	Saddle	Exists and Stable	Unstable	Towards (*x*^⁎^(*κ*),*y*^⁎^(*κ*))	*C*_4_, *C*_5_
	0 < *κ* < *R*	Saddle	Exists and Stable	Unstable	Towards (*x*^⁎^(*κ*),*y*^⁎^(*κ*))	*C*_3_
	*P* < *Q* < *κ* < *R*	Saddle	Exists and Unstable	Unstable	Towards G.A.S Limit Cycle	*C*_2_, *C*_4_, *C*_5_
	*R* < *κ*	Unstable	Ceases to Exist	Saddle	Eventual Prey Elimination	All Cases
$0<\frac{\beta }{\delta }<\alpha$	*κ* < *Q*	Saddle	Stable	Unstable	Towards (*x*^⁎^(*κ*),*y*^⁎^(*κ*))	*C*_5_
	*Q* < *κ* < *P*	Saddle	Exists and Unstable	Unstable	Towards G.A.S Limit Cycle	*C*_5_
	*P* < *κ*	Saddle	Does not exist	Stable	Approaches (*γ*,0)	*C*_3_, *C*_4_, *C*_5_

where $P=\frac{\delta -(\beta -\delta )\gamma^{2}}{\beta -\delta \alpha }$, $Q=\frac{-2{\left(x^{⁎}\right)}^{3}(\kappa )-\gamma (1-{\left(x^{⁎}\right)}^{2}(\kappa ))}{\alpha \gamma }$ and $R=\frac{\delta }{\beta -\delta \alpha }$

The terms *P*, *Q* and *R* in the table are obtained by solving the three bifurcation equations for *κ*.

Let us denote the interior equilibrium for the system (1.5) - (1.6) as $\left(x^{⁎}(\kappa ),y^{⁎}(\kappa )\right)$ since α>0 is assumed to be fixed. Thus, we have

(3.1) $$x^{⁎}(\kappa )=\sqrt{\frac{\delta -(\beta -\delta \alpha )\kappa }{\beta -\delta }}$$

(3.2) $$y^{⁎}(\kappa )=(1-\frac{x^{⁎}(\kappa )}{\gamma })(\frac{1+\alpha \kappa +{\left(x^{⁎}(\kappa )\right)}^{2}}{x^{⁎}(\kappa )})$$

From (3.1), we see that

(3.3) $${\left(x^{⁎}(\kappa )\right)}^{2}=\frac{\delta -(\beta -\delta \alpha )\kappa }{\beta -\delta }$$

From (3.3), we get

(3.4) $$\kappa =\frac{\delta -(\beta -\delta ){\left(x^{⁎}(\kappa )\right)}^{2}}{\beta -\delta \alpha }$$

Now, substituting (3.4) in (3.2), and rearranging the terms, we get the equation which would be satisfied by any interior equilibrium

(3.5) $$y^{⁎}(\kappa )=\frac{\beta }{\beta -\delta \alpha }(1-\frac{x^{⁎}(\kappa )}{\gamma })(\frac{1+{\left(x^{⁎}(\kappa )\right)}^{2}(1-\alpha )}{x^{⁎}(\kappa )})$$

The above equation represents the relation between the prey and the predator at the point of interior equilibrium. From the above discussion, we observe that based on the analysis in Srinivasu et al. (2018), the additional food is classified into two types based on quality: one is high quality additional food when $0<\alpha <\frac{\beta }{\delta }$ and other is low quality additional food when $0<\frac{\beta }{\delta }<\alpha$. High quality additional food implies that maximum growth rate of predators due to consumption of additional food is greater than their mortality rate. Analogously we can imply the significance of low quality of additional food.

We also observe that when α<1(1<α), we have $\frac{\epsilon_{1}}{h_{1}}<\frac{\epsilon_{2}}{h_{2}}$ ($\frac{\epsilon_{1}}{h_{1}}>\frac{\epsilon_{2}}{h_{2}}$), which implies that the maximum growth rate due to consumption of prey is more than (less than) the maximum growth rate due to consumption of additional food. However, in either case, the maximum growth rate of predators due to consumption of additional food is still more than the predators' natural death rate. Therefore, using the definition of quality of additional food, we say that in high quality additional food, the supplements are of superior high quality when compared to the prey if α∈(0,1) and of inferior high quality if $\alpha \in (1,\frac{\beta }{\delta })$. Thus, overall we classify additional food provided into three categories based on the quality:

1. Case (i) - Superior high quality additional food when $\alpha <1<\frac{\beta }{\delta }$
2. Case (ii) - Inferior high quality additional food when $1<\alpha <\frac{\beta }{\delta }$
3. Case (iii) - Low quality additional food when $1<\frac{\beta }{\delta }<\alpha$

**Figure 2:**
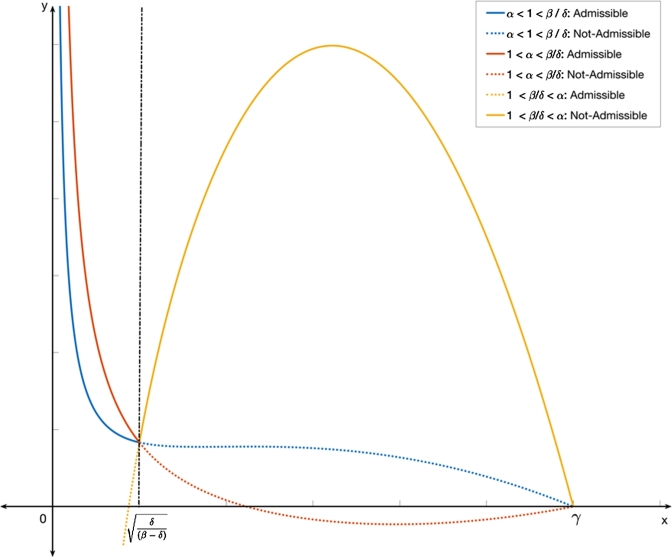
This figure depicts the prey-predator relationship at the interior equilibrium denoted by the admissible curve (3.5) for Cases (i) - (iii). In Case - (i): when superior high quality additional food is provided to the predators. The solid line in Blue color represents the admissible equilibrium points when the prey component is such that $x<\sqrt{\delta /(\beta -\delta )}$. In Case (ii) when inferior high quality additional food is provided to the predators, we obtain the equilibrium points become admissible when the prey component is such that $x<\sqrt{\delta /\beta -\delta }$. This is represented by the solid line in Red color. Note that since *α* > 1 in this case, $\sqrt{1/(\alpha -1)}>0$ and when $\sqrt{1/(\alpha -1)}<\gamma$, the curve intersects the prey axis at $x<\sqrt{\delta /(\beta -\delta )}$. In Case (iii) when low quality additional food is provided to the predators, is provided, the equilibrium points become admissible when the prey component is such that $x>\sqrt{\frac{\delta }{\beta -\delta }}$. This is represented by the solid line in Yellow color. We note that since *α* > 1 in this case, $\sqrt{1/(\alpha -1)}>0$ and when $\sqrt{1/(\alpha -1)}<\gamma$, the curve intersects the prey axis at $x<\sqrt{\delta /(\beta -\delta )}$.

Fig. 2 shows how curve representing the prey-predator relation (3.5) at the interior equilibrium behaves in each of the above mentioned cases. The curve depicted in (3.5) is obtained from the prey nullcline (or isocline) without the dependency of the parameter *κ*. The study of global dynamics of the system (1.5) - (1.6) suggests that if the system admits interior equilibrium in the absence of additional food, then $0<\overset{ˆ}{x}=\sqrt{\frac{\delta }{\beta -\delta }}<\gamma$ ($\overset{ˆ}{x}$ is the *x* - component of interior equilibrium for initial system Srinivasu et al. (2018)) and as a result, we observe from equation (3.1) that when high quality additional food is provided (i.e., in cases (i) and (ii)), only those points on the curve (1.6) lying to the left of $\overset{ˆ}{x}$ become admissible. In other words, when β−δα>0, then only those points on the curve (3.5) with $x\in [0,\sqrt{\frac{\delta }{\beta -\delta }})$ become admissible equilibrium solutions. The admissible points are depicted by the solid lines in Fig. 2.

On the other hand, if the initial system does not admit interior equilibrium, then the additional food of high quality must be provided in quantity $\kappa \in (P,R)=(\frac{\delta -\gamma^{2}(\beta -\delta )}{\beta -\delta \alpha },\frac{\delta }{\beta -\delta \alpha })$ in order to bring co-existence of predator and prey into the system. From Fig. 2, we also see that in case (i), there is a crest and trough in the equilibrium curve (curve in blue color) whereas in case (ii), the equilibrium curve is monotonically decreasing (curve in red color). This means that in the former case, there is a possibility of choosing two terminal states by the eco-manager for the same predator population level which is not the case in the latter.

When low quality additional food is provided, suppose the initial system admits interior equilibrium, then we see from the case (iii) of Fig. 2 that those points on the equilibrium curve (3.5) represented in yellow color corresponding to $x\in (\sqrt{\frac{\delta }{\beta -\delta }},\gamma ]$ become admissible. But if the initial system does not admit interior equilibrium, then the additional food system cannot bring in co-existence into the system by providing any amount of low quality of additional food.

Now, we will look into the possibility of managing pest through these admissible equilibria. From the admissible segments of the equilibrium curve (3.5) shown in Fig. 2, we see that to achieve eventual elimination of prey (pest), we must provide high quality additional food. This means that only in cases (i) and (ii), we can drive the system towards prey elimination. Also, due to the nature of the type III functional response, the complete eradication of prey (with $x^{⁎}=0$) would blow up the predator population. Thus, we consider minimal density of the prey (pest) at the terminal state in such a way that the pests do not cause any damage to the ecosystem at that density. Based on the above discussion, we now propose the following result which is relevant in the case of pest management.

Proposition 1 *If additional food provided to the predators is of high quality satisfying* β−δα>0 *, then the pest can be driven to minimal level towards elimination by provided the quantity of additional food supply satisfies* $\kappa >\frac{\delta }{\beta -\delta \alpha }$ *. Moreover, if the additional food provided is of low quality with* β−δα<0 *, then pest management cannot be achieved by providing additional food to the predators.*

Proof The prey component of the interior equilibrium is given by

$$x=\frac{\delta -(\beta -\delta \alpha )\kappa }{\beta -\delta }$$

from which we can observe that the additional food system (1.5) - (1.6) does not admit any interior equilibrium when β−δα>0 and $\kappa >\frac{\delta }{\beta -\delta \alpha }$. Also, from the dynamics presented in the Table 4, we observe that when β−δα>0 and $\kappa >\frac{\delta }{\beta -\delta \alpha }$, the trivial equilibrium is unstable and the axial equilibrium (γ,0) is a saddle whose unstable manifold moves towards origin on the prey axis.

Thus, for any solution initiated under the prey isocline curve, the saddle of axial equilibrium pushes the trajectory towards origin whose unstable nature in turn drives the system asymptotically towards the predator axis eventually eliminating the prey. On the other hand, for the solutions generating above the prey isocline curve, the prey isocline curve itself acts as an unstable manifold of the axial equilibrium thereby driving the state towards prey elimination. Therefore, we can conclude that by choosing the desired terminal state of prey to be some ϵ>0, pest management could be achieved not as an asymptote but in finite time. □

Having seen the possibility of driving the system to a state of least harmful pest, we now shift our focus to see how to achieve co-existence of species leading to the biological conservation of either of the species (or both the species). In this case, the system should be driven to the admissible segments of the curve (3.5) in all the above mentioned cases such that for the desired terminal states, there is a corresponding quantity of additional food which can sustain the system at that population after we optimally drive the system to the desired state.

**Figure 3:**
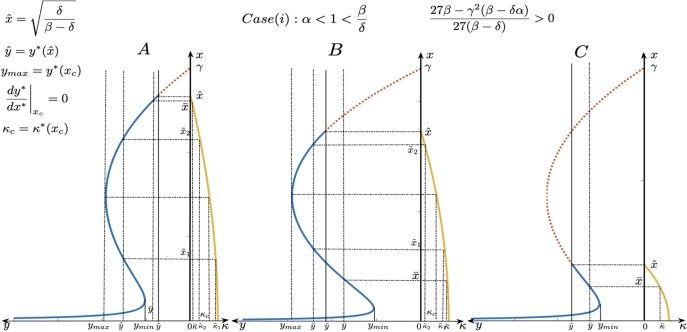
This figure contains two-quadrant graphs which represent the relationship between the admissible equilibria (solid line representing the curve (3.5)) and the quantity of additional food (3.4) when superior high quality additional food is provided and with $\frac{27\beta \gamma^{2}(\beta -\delta \alpha )}{27\beta -\delta }>0$. Thus, there exists crest and trough in the curve representing the prey-predator relation leading to possibilities of choosing two different admissible prey densities (and correspondingly two quantities of additional food depending on prey) for a given predator density.

**Figure 4:**
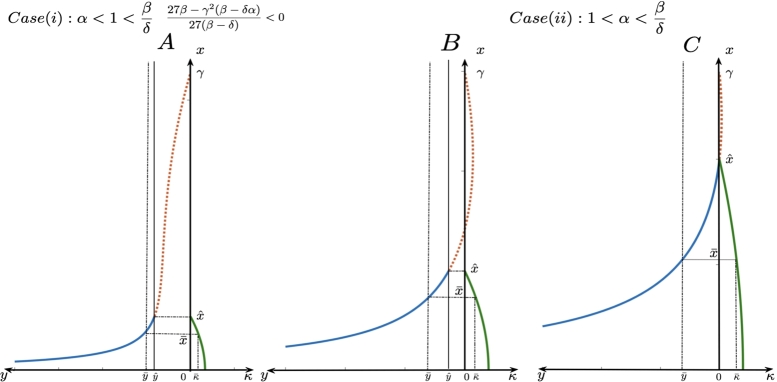
This figure contains two-quadrant graphs which represent the relationship between the admissible equilibria (solid line representing the curve (3.5)) and the quantity of additional food (3.4) where frame A represents the case when superior high quality additional food is provided and with $\frac{27\beta \gamma^{2}(\beta -\delta \alpha )}{27\beta -\delta }<0$ where as frames B and C depict the case when inferior high quality additional food is provided to the predators. We note that in both the cases, the curve representing the prey-predator relation is monotonically decreasing with increase in prey density. In these curves, we observe that for a given predator density, there exist unique admissible prey density and corresponding quantity of additional food.

**Figure 5:**
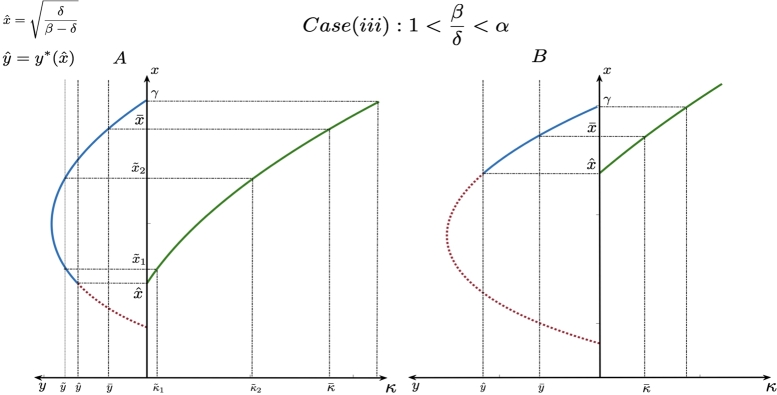
This figure contains two-quadrant graphs which represent the relationship between the admissible equilibria (solid line representing the curve (3.5)) and the quantity of additional food (3.4) where predator is provided with low quality additional food. Here too, depending on the position of $\sqrt{\delta /(\beta -\delta )}$, we can get two possibilities of admissible equilibria for a chosen predator density.

To that end, it is important to understand the nature of the prey-predator relation (3.5) and also the relation between interior equilibria and the quantity of additional food. These relations can be observed from the two-quadrant Figs. 3, 4 and 5. These have been plotted based on equations (3.3) and (3.5). We will now discuss for each case of fixed quality of additional food, the possibilities of driving the system towards the admissible equilibria (and sustaining at that state with the corresponding admissible quantity of additional food) in order to achieve biological conservation.

By observing the parameters involved in (3.5), we can conclude that the curve of interior equilibrium points is of one of the following three types:

1. The curve (3.5) has a crest and a trough for case (i) (see Fig. 3).
2. The curve (3.5) is monotonically decreasing for case (i) and case (ii) (Fig. 4).
3. The curve is concave with a hump for case (iii) (see Fig. 5).

The details regarding the nature and the behaviour of curve (3.5) and how changes in $x^{⁎}$ affect the changes in $y^{⁎}$ have been comprehensively discussed in Section 2 of the Appendix. We will now define a few terms before further discussion about the admissible equilibrium points. When the curve admits a crest and trough, the *y* - component of the point of trough is represented by $y_{min}^{⁎}$. The *y* - component of the point of crest where the curve has a hump is represented by $y_{\max}$ and the corresponding prey density and quantity are represented by $x_{c}$ and $\kappa_{c}$ respectively. The interior equilibrium of the initial system is represented by $\left(\overset{ˆ}{x},\overset{ˆ}{y}\right)$, which determines the admissibility of the interior equilibrium point on the curve (3.5).

As we saw that the curve can assume different shapes, depending on the position of $\overset{ˆ}{y}$ and the nature of the curve (3.5), we see that a desired terminal predator population $\overset{¯}{y}$ could have a unique choice of quantity of additional food $\overset{¯}{\kappa }$ that makes the corresponding state $\left(\overset{¯}{x}(\overset{¯}{\kappa }),\overset{¯}{y}\right)$ admissible or for a desired terminal predator population $\overset{˜}{y}$ there could be two choices of quantity of additional food ${\overset{¯}{\kappa }}_{1}$ and ${\overset{¯}{\kappa }}_{2}$ such that the corresponding states $\left({\overset{˜}{x}}_{1}({\overset{˜}{\kappa }}_{1}),\overset{˜}{y}\right)$ and $\left({\overset{˜}{x}}_{2}({\overset{˜}{\kappa }}_{2}),\overset{˜}{y}\right)$ both are admissible. Here, the eco-manager can choose the desired terminal state according the admissible options available that benefit the ecosystem.

The following results sum up the above discussion on admissible equilibrium states for each case of fixed quality.

Proposition 2 *If the quality of additional food satisfies the condition* $\alpha <1<\frac{\beta }{\delta }$ *and* $\sqrt{\frac{27\beta -\gamma^{2}(\beta -\delta \alpha )}{27(\beta -\delta )}}>0$ *, then*

*(i)* *For desired terminal predator population* $\min\{\overset{ˆ}{y},y_{\min}\}<\overset{¯}{y}<\min\{\overset{ˆ}{y},y_{\min}\}$ *, there exists a unique additional food quantity* $\overset{¯}{\kappa }$ *such that* $\left(\overset{¯}{x}(\overset{¯}{\kappa }),\overset{¯}{y}\right)$ *is an admissible equilibrium point for the system* (1.5) *-* (1.6) *(see* Fig. 3 *).*
*(ii)* *Suppose* $x_{c}<x_{\max}$ *, then for* $\max\{y_{\min},\overset{ˆ}{y}\}<\overset{˜}{y}<y_{\max}$ *, there exist two choices of quantity of additional food* $\kappa_{1}$ *,* $\kappa_{2}$ *such that* $\left({\overset{˜}{x}}_{1}({\overset{˜}{\kappa }}_{1}),\overset{˜}{y}\right)$ *and* $\left({\overset{˜}{x}}_{2}({\overset{˜}{\kappa }}_{2}),\overset{˜}{y}\right)$ *are admissible equilibrium points for the system* (1.5) *-* (1.6) *(see frames A and B of* Fig. 3 *).*

*On the other hand, if the quality of additional food satisfies the condition* $\alpha <1<\frac{\beta }{\delta }$ *and* $\sqrt{\frac{27\beta -\gamma^{2}(\beta -\delta \alpha )}{27(\beta -\delta )}}<0$ *, then for* $\overset{¯}{y}>\overset{ˆ}{y}$ *, there exists a unique additional food quantity* $\overset{¯}{\kappa }$ *such that* $\left(\overset{¯}{x(\overset{¯}{\kappa })},\overset{¯}{y}\right)$ *is an admissible equilibrium point for the system* (1.5) *-* (1.6) *(see frame - A of* Fig. 4 *).*

Proposition 3 *If the quality of additional food satisfies the condition* $1<\alpha <\frac{\beta }{\delta }$ *, then for the desired predator population* $\overset{¯}{y}>\overset{ˆ}{y}$ *, there exists a unique additional food quantity* $\overset{¯}{\kappa }$ *such that* $\left(\overset{¯}{x}(\overset{¯}{\kappa }),\overset{¯}{y}\right)$ *is an admissible equilibrium point for the system* (1.5) *-* (1.6) *(see frames B and C of* Fig. 4 *).*

Proposition 4 *If the quality of additional food satisfies the condition* $1<\frac{\beta }{\delta }<\alpha$ *, then*

*(i)* *For the desired predator density* $0<\overset{¯}{y}<\overset{ˆ}{y}$ *, there exists a unique additional food quantity* $\overset{¯}{\kappa }$ *such that* $\left(\overset{¯}{x}(\overset{¯}{\kappa }),\overset{¯}{y}\right)$ *is an admissible equilibrium point for the system* (1.5) *-* (1.6) *. (See* Fig. 5 *.)*
*(ii)* *Suppose* $\overset{ˆ}{x}<x_{c}$ *, then for* $\overset{ˆ}{y}<\overset{˜}{y}<y_{\max}$ *, there exist two choices of quantity of additional food* $\kappa_{1}$ *,* $\kappa_{2}$ *such that* $\left({\overset{˜}{x}}_{1}({\overset{˜}{\kappa }}_{1}),\overset{˜}{y}\right)$ *and* $\left({\overset{˜}{x}}_{2}({\overset{˜}{\kappa }}_{2}),\overset{˜}{y}\right)$ *are admissible equilibrium points for the system* (1.5) *-* (1.6) *(see frame A of* Fig. 5 *).*

## 4 Time optimal control problem

In this section, we formulate and study an optimal control problem associated with the additional food system (1.5) - (1.6) that minimizes the time to reach the desired admissible state $\left(\overset{¯}{x},\overset{¯}{y}\right)$ from any initial state $\left(x_{0},y_{0}\right)$ of the system using quantity of additional food (*κ*) as the control parameter. Further, we establish the existence of an optimal solution and also establish the characteristics of the optimal solution.

### 4.1 Formulation of control problem and existence of solution

Let us fix the quality of additional food provided α>0 to be a constant throughout this study and vary the quantity of additional food in the interval $\left[\kappa_{\min},\kappa_{\max}\right]$. We now define the Mayer's control problem of minimum time Cesari (2012) as follows:

(4.1) $$\begin{array}{c} \min_{\kappa_{\min}\leq \kappa (t)\leq \kappa_{\max}}T\\ \text{subject to:}\\ \overset{˙}{x}=f_{1}(x,y,\kappa )\\ \overset{˙}{y}=f_{2}(x,y,\kappa )\\ (x(0),y(0))=(x_{0},y_{0})\;\text{and}\;(x(T),y(T))=(\overset{¯}{x},\overset{¯}{y}), \end{array}\}\text{Optimal Control Problem}$$

where $f_{1}(x,y,\kappa )=x(1-\frac{x}{\gamma })-(\frac{x^{2}y}{1+\alpha \kappa +x^{2}})\text{ and }f_{2}(x,y,\kappa )=\beta (\frac{x^{2}+\kappa }{1+\alpha \kappa +x^{2}})y-\delta y$. The functions $f_{1}(x,y,\kappa )$ and $f_{2}(x,y,\kappa )$ can also be represented using two functions $f(x)=\frac{x}{1+\alpha \kappa +x^{2}}$ and $g(x)=(1-\frac{x}{\gamma })(1+\alpha \kappa +x^{2})$ as follows

(4.2) $$f_{1}(x,y,\kappa )=(g(x)-xy)f(x)$$

(4.3) $$f_{2}(x,y,\kappa )=[\beta f(x)(x+\frac{\kappa }{x})-\delta ]y$$

This optimal control problem (4.1) is a *Mayer time optimal control problem* (Section 3, of the Appendix) with n=2, m=1 and x(t)=(x(t),y(t)), u(t)=κ(t) with $\text{f}(t,\text{x}(t),\text{u}(t))=(f_{1}(x,y,\kappa ),f_{2}(x,y,\kappa ))$. The boundary conditions are $e[\text{x}]=(0,x_{0},y_{0},T,\overset{¯}{x},\overset{¯}{y})$.

The set *A* associated with the control problem (4.1) is the subset of *t***x** - space ($R^{1+2}$), i.e., $A\subset R^{1+2}$ from which we obtain the state variables. The set of all admissible solutions of the control problem (4.1) which is a subset of *A* can be defined as

$$\Omega :=\{(\text{x},\kappa )=(x,y,\kappa ):(\text{x},\kappa )\text{ is an admissible pair for the problem}\;\text{(4.1) with }\kappa \;\in [\kappa_{\min},\kappa_{\max}]\}$$

Now, we want to obtain a solution from the set Ω which minimizes the time to reach the terminal state (x(T),y(T)) which would be the optimal solution for (4.1). We will establish this in the following theorem by proving the existence of an optimal control using *Filippov's Existence Theorem* (refer to Section 3 of the Appendix) which drives the system to a desired terminal state in minimum time.

Theorem 1 *If the desired terminal state of the system*$\left(\overset{¯}{x},\overset{¯}{y}\right)$*satisfies the conditions in*Proposition 1, Proposition 4*(that make the terminal state admissible) depending on the quality of additional food, then there exists an optimal control*$\kappa^{⁎}(t)$*that drives the system from an initial state*$\left(x_{0},y_{0}\right)$*to the desired terminal state*$\left(\overset{¯}{x},\overset{¯}{y}\right)$*in minimum (finite) time for the time optimal control problem*(4.1)*provided the set of admissible solutions* Ω *is non-empty.*

Proof To prove this theorem, we will use the *Filippov's Existence Theorem* and show that all the conditions in the hypothesis of the theorem are satisfied. This will be sufficient to show that the considered optimal control problem (4.1) has an optimal solution. Thus, we need to establish that the following conditions are satisfied:

1. The set A is compact.
2. The set of all controls $\left[\kappa_{\min},\kappa_{\max}\right]$ is compact.
3. The set of boundary points $B=\{0,x_{0},y_{0},T,\overset{¯}{x},\overset{¯}{y}\}$ is compact and objective function is continuous on *B*.
4. For every (x,y)∈A the sets $Q(x,y):=\{(z_{1},z_{2})|z_{1}=f_{1}(x,y,\kappa ),z_{2}=f_{2}(x,y,\kappa ),\kappa \;\in \;[\kappa_{\min},\kappa_{\max}]\}$ are convex.

Let us consider each of the above conditions and show that the optimal control problem (4.1) satisfies all of them.

(i) From the global dynamics of the system (1.5) - (1.6) described in the Table 4, we see that whenever βκ−δ(1+δκ)<0, the solution trajectories that are generated reach the predator axis eventually. Using the Proposition 1, we see that by considering x(T)=ϵ which is sufficiently small, the terminal state $\left(\epsilon ,y^{⁎}(\epsilon )\right)$ can be reached in finite time unlike reaching x(T)=0. Thus, in this case, the solution space is closed and bounded. On the other hand, whenever βκ−δ(1+δκ)>0, the positivity and boundedness result established in Section 4 of the Appendix shows that the solutions are closed and bounded. Thus, we can conclude that the set *A* is compact and this proves condition 1.
(ii) Conditions 2 and 3 are satisfied from the definitions of the respective sets $\left[\kappa_{\min},\kappa_{\max}\right]$ and $B=\{0,x_{0},y_{0},T,\overset{¯}{x},\overset{¯}{y}\}$ and also by definition of the objective function J[κ]=T.
(iii) To prove condition 4, we need to show that the sets Q(x,y) are convex. To that end, consider $z_{1}=f_{1}(x,y,\kappa )=x(1-\frac{x}{\gamma })-(\frac{x^{2}y}{1+\alpha \kappa +x^{2}})$. Rearranging the terms, we get
  $$(\frac{x^{2}y}{1+\alpha \kappa +x^{2}})=x(1-\frac{x}{\gamma })-z_{1}$$
  Cancelling the extra terms, we get
  (4.4) $$(\frac{y}{1+\alpha \kappa +x^{2}})=(1-\frac{x}{\gamma })-\frac{z_{1}}{x}$$
  Now consider
  $$z_{2}=f_{2}(x,y,\kappa )=\beta (\frac{x^{2}+\kappa }{1+\alpha \kappa +x^{2}})y-\delta y$$
  Replacing the term $\frac{y}{1+\alpha \kappa +x^{2}}$ in the above expression using equation (4.4), we get
  $$z_{2}=\beta (x^{2}+\kappa )((1-\frac{x}{\gamma })-\frac{z_{1}}{x})-\delta y=-\beta (x+\frac{\kappa }{x})z_{1}+\beta (x^{2}+\kappa )(1-\frac{x}{\gamma })-\delta y$$
  By rearranging the last expression, we get
  (4.5) $$\beta (x+\frac{\kappa }{x})z_{1}+z_{2}=\beta (x+\kappa )(1-\frac{x}{\gamma })-\delta y$$
  From the equation (4.5), we see that the sets Q(x,y) are linear segments of its components which are convex. This proves Condition 4.

Hence, the time optimal control problem (4.1) admits an optimal solution provided the set of admissible solutions Ω is non-empty. □

### 4.2 Characteristics of optimal solution

In this subsection, we will assume that the optimal solution exists and obtain the characteristics of the optimal solution trajectory using the *Pontryagin's Maximum Principle* Liberzon (2011).

Firstly, we define the Hamiltonian function associated with the control problem (4.1) as

$$H(x,y,\kappa ,\lambda ,\mu ):=\lambda \frac{dx}{dt}+\mu \frac{dy}{dt}$$

where, *λ* and *μ* are called the Adjoint variables or Co-state variables. Substituting for $\frac{dx}{dt}$ and $\frac{dx}{dt}$ from the system equations (1.5) - (1.6), the Hamiltonian becomes

(4.6) $$H(x,y,\kappa ,\lambda ,\mu )=\lambda [x(1-\frac{x}{\gamma })-(\frac{x^{2}y}{1+\alpha \kappa +x^{2}})]+\mu [\beta (\frac{\left(x^{2}+\kappa \right)}{1+\alpha \kappa +x^{2}})y-\delta y]$$

By rearranging the equation (4.6), we get

(4.7) $$H(x,y,\kappa ,\lambda ,\mu )=(\lambda x(1-\frac{x}{\gamma })-\mu \delta y)-\frac{y}{1+\alpha \kappa +x^{2}}(\lambda x^{2}-\mu \beta (x^{2}+\kappa ))$$

Now, using the representation for $f_{1}(x,y,\kappa )$ and $f_{2}(x,y,\kappa )$ in the equations (4.2) - (4.3), the Hamiltonian can be represented as

(4.8) $$H(x,y,\kappa ,\lambda ,\mu )=\lambda [(g(x)-xy)f(x)]+\mu [\beta f(x)(x+\frac{\kappa }{x})-\delta ]y$$

The Maximum Principle states that if an optimal solution exists, then the Co-state variables must satisfy the canonical equations (adjoint system) given by $\frac{d\lambda }{dt}=-\frac{\partial H}{\partial x},\;\frac{d\mu }{dt}=-\frac{\partial H}{\partial y}$. Using the equation (4.8), the adjoint system can be written as

(4.9) $$\frac{d\lambda }{dt}=-\lambda \{[g_{x}(x,\alpha ,\kappa )-y]f(x,\alpha ,\kappa )+(g(x,\alpha ,\kappa )-xy)f_{x}(x,\alpha ,\kappa )\}-\mu \beta y\{f_{x}(x,\alpha ,\kappa )(x+\frac{\kappa }{x})+(1-\frac{\kappa }{x^{2}})f(x,\alpha ,\kappa )\}$$

(4.10) $$\frac{d\mu }{dt}=\lambda f(x,\alpha ,\kappa )-\mu \{\beta f(x,\alpha ,\kappa )(x+\frac{\kappa }{x}-\delta \}$$

Now, we differentiate the Hamiltonian function (4.7) with the control variable *κ* to obtain the characteristics of the optimal control solution.

(4.11) $$\frac{\partial H}{\partial \kappa }=\frac{y}{{\left(1+\alpha \kappa +x^{2}\right)}^{2}}(\lambda \alpha x^{2}+\mu \beta [1+x^{2}(1-\alpha )])$$

Since the above equation (4.11) cannot give $\kappa^{⁎}(t)$ in a closed form, we differentiate the Hamiltonian function the second time with respect to *κ* and we get

(4.12) $$\frac{\partial^{2}H}{\partial \kappa^{2}}=-(\frac{2\alpha }{1+\alpha \kappa +x^{2}})\frac{\partial H}{\partial \kappa }$$

The above expression implies that the Hamiltonian function is either monotone increasing function or monotone decreasing function with respect to the variable *κ* provided $\frac{\partial H}{\partial \kappa }\neq 0$. Since our control problem (4.1) is a minimization problem, the Hamiltonian maximization condition Liberzon (2011) becomes a minimization condition in our scenario according to which we get that

(4.13) $$H(x^{⁎}(t),y^{⁎}(t),\kappa^{⁎}(t),\lambda^{⁎}(t),\mu^{⁎}(t))\leq H(x^{⁎}(t),y^{⁎}(t),\kappa ,\lambda^{⁎}(t),\mu^{⁎}(t))$$

$\forall \kappa \in [\kappa_{\min},\kappa_{\max}]$ and ∀t∈[0,T]. Also, since (4.1) is a time optimal control problem, it can be proved that the Hamiltonian function is a constant along the optimal trajectory and in particular, it assumes value -1 Clark (1974). Hence,

(4.14) H(x(t),y(t),κ,λ(t),μ(t))=−1

Now, using the expression for Hamiltonian (4.7), using the Hamiltonian minimization condition (4.13), and also the monotonicity property of the Hamiltonian function with respect to *κ*, we can conclude that the optimal control might be of bang-bang type provided no singular solution exists for a sub-interval in [0,T]. This implies that optimal control function would assume the form:

$$\alpha^{⁎}(t)=\left\{ \begin{array}{cc} \kappa_{\max}, & \text{ if}\;\frac{\partial H}{\partial \kappa }<0\\ \kappa_{\min}, & \text{ if}\;\;\frac{\partial H}{\partial \kappa }>0\\ ? & \text{ if}\;\;\frac{\partial H}{\partial \kappa }=0 \end{array}\right.$$

When $\frac{\partial H}{\partial \kappa }=0$ for any sub-interval of [0,T], the optimal control function cannot be obtained using the Hamiltonian minimization condition and the monotonicity property. This is called singularity in optimal solution. To show that the optimal solution is of bang-bang type only, we must show that the solution does not exhibit singular arc in any interval $\left(t_{1},t_{2}),\subseteq [0,T\right]$. Thus, to know the characterize exactly the optimal control, we assume that singular solution exists and analyse the optimal solution.

Suppose the singular solution occurs at some time t∈[0,T], then $\frac{\partial H}{\partial \kappa }{|}_{t}=0$. This means that

(4.15) $$\lambda \alpha x^{2}+\mu \beta [1+x^{2}(1-\alpha )]=0$$

rearranging which we get

(4.16) $$\frac{\lambda }{\mu }=\frac{\beta [x^{2}(\alpha -1)-1]}{\alpha x^{2}}$$

The above equation implies that along singular solution the co-state variables behave as follows:

(i) *λ* and *μ* will be of opposite signs when $x<\sqrt{\frac{1}{\alpha -1}}$.
(ii) *λ* and *μ* will be of same sign when $x>\sqrt{\frac{1}{\alpha -1}}$.
(iii) λ=0 and *μ* will be arbitrary when $x=\sqrt{\frac{1}{\alpha -1}}$

The behaviour of the co-state variables along the singular solution shows that both of them cannot become zero simultaneously as that would contradict the equation (4.14) along the optimal solution. To characterize the optimal trajectory along the singular solution, we differentiate the equation (4.11) with respect to time. This gives us

$$\frac{d}{dt}\frac{\partial H}{\partial \kappa }=\frac{d}{dt}[\frac{y}{{\left(1+\alpha \kappa +x^{2}\right)}^{2}}(\lambda \alpha x^{2}+\mu \beta [1+x^{2}(1-\alpha )])]=0=(\lambda \alpha x^{2}+\mu \beta [1+x^{2}(1-\alpha )])\frac{d}{dt}(\frac{y}{{\left(1+\alpha \kappa +x^{2}\right)}^{2}})+(\frac{y}{{\left(1+\alpha \kappa +x^{2}\right)}^{2}})\frac{d}{dt}(\lambda \alpha x^{2}+\mu \beta [1+x^{2}(1-\alpha )])=0$$

Using the fact that equation (4.15) holds along singular solution, the above expression becomes

(4.17) $$\frac{d}{dt}\frac{\partial H}{\partial \kappa }=(\frac{y}{{\left(1+\alpha \kappa +x^{2}\right)}^{2}})\frac{d}{dt}(\lambda \alpha x^{2}+\mu \beta [1+x^{2}(1-\alpha )])=0$$

Let us expand the expression on the right hand side of the above equation

(4.18) $$\frac{d}{dt}(\lambda \alpha x^{2}+\mu \beta [1+x^{2}(1-\alpha )])=\alpha x^{2}\frac{d\lambda }{dt}+\beta [1+(1-\alpha )x^{2}]\frac{d\mu }{dt}+2[\lambda \alpha x+\mu \beta x(1-\alpha )]\frac{dx}{dt}$$

Expanding the terms in the above expression using the additional food system (1.5) - (1.6), the adjoint system (4.9) - (4.10) and simplifying the terms using equation (4.15) along the singular solution, the equation (4.18) becomes

(4.19) $$\frac{d}{dt}(\lambda \alpha x^{2}+\mu \beta [1+x^{2}(1-\alpha )])=\frac{\mu \beta }{\gamma \alpha }[2\alpha (\alpha -1)x^{3}+\gamma (\alpha -1)[\beta -\alpha (\delta +1)]x^{2}-\gamma (\beta -\delta \alpha )]$$

Substituting (4.19) in (4.17), we get

(4.20) $$\frac{d}{dt}\frac{\partial H}{\partial \kappa }=(\frac{\mu \beta y}{\gamma \alpha {\left(1+\alpha \kappa +x^{2}\right)}^{2}})[2\alpha (\alpha -1)x^{3}+\gamma (\alpha -1)[\beta -\alpha (\delta +1)]x^{2}-\gamma (\beta -\delta \alpha )]=0$$

The above equation implies that along the singular solution, we have

(4.21) $$2\alpha (\alpha -1)x^{3}+\gamma (\alpha -1)[\beta -\alpha (\delta +1)]x^{2}-\gamma (\beta -\delta \alpha )=0$$

which means that if singularity occurs in the optimal solution of the control problem (4.1), then it occurs at the roots of the above cubic equation (provided the roots are real and positive). To get more insight into the points of singularity, we differentiate equation (4.20) again with respect to time along singular solution assuming that at least one root of the cubic equation (4.21) is real and positive (denoted by $\overset{ˆ}{x}$). Then, using equation (4.15) we get

(4.22) $$\frac{d^{2}}{dt^{2}}\frac{\partial H}{\partial \kappa }=(\frac{\mu \beta y}{\gamma \alpha {\left(1+\alpha \kappa +x^{2}\right)}^{2}})[6\alpha (\alpha -1){\overset{ˆ}{x}}^{2}+2\gamma (\alpha -1)[\beta -\alpha (\delta +1)]\overset{ˆ}{x}]\times (g(\overset{ˆ}{x},\alpha ,\kappa )-\overset{ˆ}{x}y)f(\overset{ˆ}{x},\alpha ,\kappa )=0$$

from which we can conclude that if singularity occurs in the optimal control solution of the control problem (4.1) and if at least one of the roots of the cubic equation (4.21) denoted by $\overset{ˆ}{x}$ is real and positive, then the *y* component of the point in the solution space where singularity occurs is given by

(4.23) $$\overset{ˆ}{y}=\frac{g(\overset{ˆ}{x},\alpha ,\kappa )}{\overset{ˆ}{x}}$$

Thus, the singularity, if occurs, will be at points $\left(\overset{ˆ}{x},\overset{ˆ}{y}\right)$ and there is no possibility for a singular arc. Thus we can conclude that the optimal control can be established using the Hamiltonian minimization condition and monotonicity property as guessed above. Summarizing the above analysis we will now state a result that characterizes the optimal solution of the control problem (4.1)

Theorem 2 *The optimal control strategy for the time optimal control problem* (4.1) *is a combination of bang-bang controls only, with possibility of switches occurring at specific points in the optimal trajectory. The Optimal control is given by*

(4.24) $$\kappa^{⁎}(t)=\left\{ \begin{array}{cc} \kappa_{\max}, & \;\text{if}\;\;\frac{\partial H}{\partial \kappa }<0\\ \kappa_{\min}, & \;\text{if}\;\;\frac{\partial H}{\partial \kappa }>0 \end{array}\right.$$

Now, based on the theorem stated above, we state a corollary to the existence theorem (Theorem 1).

Corollary 1 *If an admissible path connecting the initial state* $\left(x_{0},y_{0}\right)$ *and the terminal state* $\left(\overset{¯}{x},\overset{¯}{y}\right)$ *involving a combination of bang-bang controls exists, then the optimal control problem* (4.1) *has an optimal solution.*

## 5 Nature of optimal solution trajectories and applications to pest management

In this section, we study the properties of optimal solutions and switch points and analyse the biological relevance of the outcomes. To that end, consider the following equation whose roots determine the points of singularity in the optimal trajectory:

(5.1) $$F(x)\equiv 2\alpha (\alpha -1)x^{3}+\gamma (\alpha -1)[\beta -\alpha (\delta +1)]x^{2}-\gamma (\beta -\delta \alpha )=0$$

We want understand the nature of the equation to be able to determine the number of roots that would exist depending on the nature of parameters. Using the Descarte's Rule of Signs (as was used for understanding the nature of the curve (3.5) in Section 2 of the Appendix), we obtain the following conclusions about the equation (5.1) and the phase space of the solution trajectory of (4.1) in each of the cases (i) - (iv) of fixed quality:

1. Case (i) - $\alpha <1<\frac{\beta }{\delta }$: In this case, we see that as *x* increases, F(x)→−∞ and as *x* decreases, F(x)→+∞. Also, using the rule of signs, we can show that equation (5.1) certainly has one negative root. The remaining roots depend on the term β−α(δ+1) and the nature of the function $\frac{dF(x)}{dx}$ and we can see that $\frac{dF(x)}{dx}=0$ implies that $x_{1}=0$ and $x_{2}=\frac{\gamma [\beta -\alpha (\delta +1)]}{3\alpha }$ where the curve (5.1) changes its nature and correspondingly, $F(x_{1})<0$ and $F(x_{2})<0$. Thus, we can conclude that in this case the remaining two are not positive and hence the phase space can be divided into the following regions:
  $$\text{Region Ia}:=\{(x,y)\;|\;y<\frac{\beta }{\beta -\delta \alpha }(1-\frac{x^{⁎}(\kappa )}{\gamma })(\frac{1+{\left(x^{⁎}(\kappa )\right)}^{2}(1-\alpha )}{x^{⁎}(\kappa )})\}\text{Region Ib}:=\{(x,y)\;|\;y>\frac{\beta }{\beta -\delta \alpha }(1-\frac{x^{⁎}(\kappa )}{\gamma })(\frac{1+{\left(x^{⁎}(\kappa )\right)}^{2}(1-\alpha )}{x^{⁎}(\kappa )})\}$$
2. Case (ii) - $1<\alpha <\frac{\beta }{\delta }$: In this case, since α>1, we see that as *x* increases, F(x)→+∞ and as *x* decreases, F(x)→−∞. By using the rule of signs we can show that there exists one positive root $\overset{ˆ}{x}>0$ for the equation (5.1). Also, we see that the for critical points $x_{1}=0$ and $x_{2}=\frac{\gamma [\beta -\alpha (\delta +1)]}{3\alpha }$, $F(x_{1})<0$ and $F(x_{2})<0$. Thus, the remaining two roots are not positive and thus we can divide the phase space into four regions as follows:
  $$\text{Region IIa}:=\{(x,y)\;|\;y<\frac{\beta }{\beta -\delta \alpha }(1-\frac{x^{⁎}(\kappa )}{\gamma })(\frac{1+{\left(x^{⁎}(\kappa )\right)}^{2}(1-\alpha )}{x^{⁎}(\kappa )})\;\text{and}\;x<\overset{ˆ}{x}\}\text{Region IIb}:=\{(x,y)\;|\;y<\frac{\beta }{\beta -\delta \alpha }(1-\frac{x^{⁎}(\kappa )}{\gamma })(\frac{1+{\left(x^{⁎}(\kappa )\right)}^{2}(1-\alpha )}{x^{⁎}(\kappa )})\;\text{and}\;x>\overset{ˆ}{x}\}\text{Region IIc}:=\{(x,y)\;|\;y>\frac{\beta }{\delta }\frac{\beta }{\beta -\delta \alpha }(1-\frac{x^{⁎}(\kappa )}{\gamma })(\frac{1+{\left(x^{⁎}(\kappa )\right)}^{2}(1-\alpha )}{x^{⁎}(\kappa )})\;\text{and}\;x>\overset{ˆ}{x}\}\text{Region IId}:=\{(x,y)\;|\;y>\frac{\beta }{\beta -\delta \alpha }(1-\frac{x^{⁎}(\kappa )}{\gamma })(\frac{1+{\left(x^{⁎}(\kappa )\right)}^{2}(1-\alpha )}{x^{⁎}(\kappa )})\;\text{and}\;x<\overset{ˆ}{x}\}$$
3. Case (iii) - $1<\frac{\beta }{\delta }<\alpha$: In this case too, since α>1, we see that as *x* increases, F(x)→+∞ and as *x* decreases, F(x)→−∞ and also we can show that there exists one positive root $\overset{ˆ}{x}>0$ for the equation (5.1). However, for the critical points $x_{1}=0$ and $x_{2}=\frac{\gamma [\beta -\alpha (\delta +1)]}{3\alpha }$, we see that $F(x_{1})>0$ and $F(x_{2})<0$. So, we conclude that the remaining roots must be of opposite signs. Let the positive root be denoted by $\overset{¯}{x}$. Since F(x) is decreasing as *x* increases from $x_{1}$ to $x_{2}$, and from the understanding of nature of the curve (3.5) that it is convex with a hump meeting the prey axis at $x=\sqrt{\frac{1}{\alpha -1}}$ and x=γ we see that $\overset{¯}{x}<\sqrt{\frac{1}{\alpha -1}}$. Thus, in this case, we can divide the phase space into five regions:
  $$\text{Region IIIa}:=\{(x,y)\;|\;y<\frac{\beta }{\beta -\delta \alpha }(1-\frac{x^{⁎}(\kappa )}{\gamma })(\frac{1+{\left(x^{⁎}(\kappa )\right)}^{2}(1-\alpha )}{x^{⁎}(\kappa )})\;\text{and}\;\overset{¯}{x}<x<\overset{ˆ}{x}\}\text{Region IIIb}:=\{(x,y)\;|\;y<\frac{\beta }{\beta -\delta \alpha }(1-\frac{x^{⁎}(\kappa )}{\gamma })(\frac{1+{\left(x^{⁎}(\kappa )\right)}^{2}(1-\alpha )}{x^{⁎}(\kappa )})\;\text{and}\;\overset{¯}{x}<\overset{ˆ}{x}<x\}\text{Region IIIc}:=\{(x,y)\;|\;y>\frac{\beta }{\beta -\delta \alpha }(1-\frac{x^{⁎}(\kappa )}{\gamma })(\frac{1+{\left(x^{⁎}(\kappa )\right)}^{2}(1-\alpha )}{x^{⁎}(\kappa )})\;\text{and}\;x<\overset{¯}{x}<\overset{ˆ}{x}\}\text{Region IIId}:=\{(x,y)\;|\;y>\frac{\beta }{\beta -\delta \alpha }(1-\frac{x^{⁎}(\kappa )}{\gamma })(\frac{1+{\left(x^{⁎}(\kappa )\right)}^{2}(1-\alpha )}{x^{⁎}(\kappa )})\;\text{and}\;\overset{¯}{x}<x<\overset{ˆ}{x}\}\text{Region IIIe}:=\{(x,y)\;|\;y>\frac{\beta }{\beta -\delta \alpha }(1-\frac{x^{⁎}(\kappa )}{\gamma })(\frac{1+{\left(x^{⁎}(\kappa )\right)}^{2}(1-\alpha )}{x^{⁎}(\kappa )})\;\text{and}\;\overset{¯}{x}<\overset{ˆ}{x}<x\}$$
4. Case (iv) - α=1: In this case, F(x)=−γ(β−δ) which is a constant negative value. Thus, we see that there are no positive roots here too and similar to first case, the phase space can be divided into two regions.

We now state a result that describes the way switching occurs in an optimal solution trajectory.

Proposition 5 *The optimal control solution* $\kappa^{⁎}(t)$ *along the optimal path can switch from* $\kappa_{\min}$ *to* $\kappa_{\max}$ *(or* $\kappa_{\max}$ *to* $\kappa_{\min}$ *) in Regions Ia, IIa, IId, IIIb and IIId (or in Regions Ib, IIb, IIc, IIIa, IIIc and IIIe) only.*

Proof From the discussion in the previous section, we know that if optimal solution undergoes a switch, then at that instant we must have $\frac{\partial H}{\partial \kappa }=0$. From equation (4.11), this means that

$$\lambda (t)\alpha {\left(x(t)\right)}^{2}+\beta \mu (t)[1+{\left(x(t)\right)}^{2}(1-\alpha )]=0$$

Let the t=τ denote the time instant at which switching occurs. Then we get

(5.2) $$\lambda (\tau )\alpha {\left(x(\tau )\right)}^{2}+\beta \mu (\tau )[1+{\left(x(\tau )\right)}^{2}(1-\alpha )]=0$$

Also, along the optimal trajectory, we know that equation (4.14) is satisfied. Thus, even at the point of switch, we have

H(x(τ),y(τ),κ(τ),λ(τ),μ(τ))=−1

Using the definition of Hamiltonian from equation (4.7), we get

(5.3) $$(\lambda (\tau )x(\tau )(1-\frac{x(\tau )}{\gamma })-\mu (\tau )\delta y(\tau ))\;-\frac{y(\tau )}{1+\alpha \kappa (\tau )+{\left(x(\tau )\right)}^{2}}(\lambda (\tau ){\left(x(\tau )\right)}^{2}-\mu (\tau )\beta ({\left(x(\tau )\right)}^{2}+\kappa (\tau )))=-1$$

From equation (5.2), we get

(5.4) $$\lambda (\tau )x(\tau )=-\frac{\beta }{\alpha }\mu (\tau )[1+{\left(x(\tau )\right)}^{2}(1-\alpha )]$$

Substituting (5.4) in (5.3) and simplifying, we get

$$\lambda (\tau )x(\tau )(1-\frac{x(\tau )}{\gamma })-\mu (\tau )\delta y(\tau )+\frac{\beta \mu (\tau )y(\tau )}{\alpha }=-1$$

Since μ≠0 along the optimal solution, multiplying both sides of the above equation by $\frac{\alpha }{\mu (\tau )}$ and using the expression for $\frac{\lambda (\tau )}{\mu (\tau )}$ along optimal solution, we get

(5.5) $$\frac{\beta [{\left(x(\tau )\right)}^{2}(\alpha -1)-1]}{x(\tau )}(1-\frac{x(\tau )}{\gamma })+(\beta -\delta \alpha )y(\tau )=\frac{\alpha }{\mu (\tau )}$$

Rearranging the above expression, we get

(5.6) $$\mu (\tau )=-\frac{\alpha }{\left(\beta -\delta \alpha )[y(\tau )-\frac{\beta }{\beta -\delta \alpha }(1-\frac{x(\tau )}{\gamma })(\frac{1+{\left(x(\tau )\right)}^{2}(1-\alpha )}{x(\tau )})\right]}$$

From the nature of the curve (3.5) and the definition of various regions, we see that μ(τ) is positive (negative) in the regions Ia,IIa,IId,IIIa,IIIb and IIId (Ib,IIb,IIc,IIIa,IIIc,and IIIe).

Now, let $\sigma (t)=\lambda (t)\alpha {\left(x(t)\right)}^{2}+\beta \mu (t)[1+{\left(x(t)\right)}^{2}(1-\alpha )]$ and consider

$$\frac{d\sigma }{dt}=\frac{d}{dt}(\lambda (t)\alpha {\left(x(t)\right)}^{2}+\beta \mu (t)[1+{\left(x(t)\right)}^{2}(1-\alpha )])$$

Using the equations (4.18), (4.19) and using the fact that σ(τ)=0, we get

(5.7) $$\frac{d\sigma }{dt}{|}_{t=\tau }=\frac{\mu \beta }{\gamma \alpha }[2\alpha (\alpha -1)x^{3}+\gamma (\alpha -1)[\beta -\alpha (\delta +1)]x^{2}-\gamma (\beta -\delta \alpha )]{|}_{t=\tau }$$

From the optimal control strategy (4.24), we see that when the control switches from $\kappa_{\max}$ to $\kappa_{\min}$ (or $\kappa_{\min}$ to $\kappa_{\max}$) at t=τ, then $\frac{\partial H}{\partial \kappa }$ accordingly increases from negative to positive (positive to negative). Thus, $\frac{d\sigma }{dt}>0\;(<0)$ at t=τ for the switch $\kappa_{\max}$ to $\kappa_{min}$ (or $\kappa_{\min}$ to $\kappa_{\max}$). Using observations along with the equations (5.6) and (5.7) we can conclude that the switch $\kappa_{\max}$ to $\kappa_{\min}$ ($\kappa_{\min}$ to $\kappa_{\max}$) can occur in Ia,IIa,IId,IIIa,IIIb and IIId (Ib,IIb,IIc,IIIa,IIIc,and IIIe) only. □

The result proved above shows that to reach an interior point in the phase space, the optimal control solution could involve multiple switches only between extremal values $\left(\kappa_{\min}\right)$ and $\left(\kappa_{\max}\right)$. In other words, to achieve biological conservation, the additional food supply could involve switches in the amount of food that is supplied in order to ensure co-existence of species. Now, we will look at the relevance of the mathematical analysis done so far in the case of pest management.

We see from the existence theorem (Theorem 1) that for pest management scenario, in order to reach the desired state at finite time, we consider the terminal prey density as x(T)=ϵ which is a sufficiently small level such that the pest no longer damages the ecosystem and eventually they can be eliminated. Now, considering the desired terminal state to be (ϵ,y(T)), we state two important results that establish the characteristics of the co-state variables at the terminal time t=T and the characteristics of the optimal control throughout the optimal trajectory leading to pest management.

Lemma 1 *Let* β−δα>0 *and* x(T)=ϵ *. Then the time optimal control problem* (4.1) *admits an optimal solution if* $\kappa_{\max}>\frac{\delta }{\beta -\delta \alpha }$ *. Moreover, if* $0>\lambda (T)>\frac{-1}{\epsilon }$ *at the final time* t=T *, then* $\kappa^{⁎}(T)=\kappa_{opt}(T)=\kappa_{\max}$ *with* μ(T)<0 *.*

Proof Let $\kappa_{\max}>\frac{\delta }{\beta -\delta \alpha }$. Then, from the analysis presented in the Table 4 we can deduce that the system eventually tends towards pest eradication (prey elimination). With x(T)=ϵ, we see that y(T)=y(ϵ) does not become unbounded and as a result, instead of reaching eventually, the state $\left(\epsilon ,y^{⁎}(\epsilon )\right)$ is reached in finite time. Hence, for the control problem (4.1), Ω≠ϕ. Now, using the Existence theorem (Theorem 1), we conclude that there exists an optimal solution to the control problem (4.1).

Now, since the terminal state is $\left(\epsilon ,y^{⁎}(\epsilon )\right)$, according to our earlier analysis, we must have $\kappa^{⁎}(T)>\frac{\delta }{\beta -\delta \alpha }$. Using this and the equation (4.14) along the optimal trajectory, at the terminal state, we have

$$\lambda (T)\epsilon +\mu (T)(\frac{\beta \kappa^{⁎}(T)}{1+\alpha \kappa^{⁎}(T)}-\delta )y(T)=-1\;\mu (T)(\frac{\beta \kappa^{⁎}(T)}{1+\alpha \kappa^{⁎}(T)}-\delta )=-1-\lambda (T)\epsilon$$

Rearranging the terms above, we get

(5.8) $$\mu (T)=-\frac{1+\lambda (T)\epsilon }{\left(\frac{\beta \kappa^{⁎}(T)}{1+\alpha \kappa^{⁎}(T)}-\delta )y^{⁎}(\epsilon \right)}$$

When $\lambda (T)>\frac{-1}{\epsilon }$, we get 1+λ(T)ϵ>0. Also, since $\kappa^{⁎}(T)>\frac{\delta }{\beta -\delta \alpha }$, we have $\frac{\beta \kappa^{⁎}(T)}{1+\alpha \kappa^{⁎}(T)}-\delta >0$ implying that μ(T)<0. Now, at t=T, we get

$$\lambda (T)\alpha \epsilon^{2}+\mu (T)\beta [1+\epsilon^{2}(1-\alpha )]=\mu (T)\beta <0$$

which implies that

$$\frac{\partial H}{\partial \kappa }{|}_{t=T}=(\frac{y(T)}{{\left(1+\alpha \kappa^{⁎}(T)+\epsilon^{2}\right)}^{2}})(\lambda (T)\alpha \epsilon^{2}+\mu (T)\beta [1+\epsilon^{2}(1-\alpha )])=(\frac{y(T)}{{\left(1+\alpha \kappa^{⁎}(T)\right)}^{2}})(\mu (T)\beta )<0$$

Therefore, from Theorem 2, we get that $\kappa_{opt}(T)=\kappa^{⁎}(T)=\kappa_{\max}$. This proves the lemma. □

Theorem 3 *If* $\kappa_{\max}>\frac{\delta }{\beta -\delta \alpha }$ *, then the solution to the optimal control problem* (4.1) *with the terminal state* $\left(x(T),y(T))=(\epsilon ,y^{⁎}(\epsilon )\right)$ *is given by* $\kappa_{opt}(t)=\kappa^{⁎}(t)=\kappa_{\max}\;\forall t\in [0,T]$ *.*

Proof To prove this theorem, we use the zero solution of the linear system of the co-state variables (4.9) - (4.10) which can be written in matrix form as:

(5.9) $$\left(\begin{array}{c} \frac{d\lambda }{dt}\\ \frac{d\mu }{dt} \end{array}\right)=\left(\begin{array}{cc} -a_{1}(t) & -b_{1}(t)\\ a_{2}(t) & -b_{2}(t) \end{array}\right)\left(\begin{array}{c} \lambda (t)\\ \mu (t) \end{array}\right)$$

where

$$a_{1}(t)=1-\frac{2x}{\gamma }-\frac{2xy(1+\alpha \kappa )}{1+\alpha \kappa +x^{2}}\;b_{1}(t)=\frac{2\beta xy(1+\alpha \kappa -\kappa )}{{\left(1+\alpha \kappa +x^{2}\right)}^{2}}\;a_{2}(t)=\frac{-x^{2}}{1+\alpha \kappa +x^{2}}\;b_{2}(t)=\frac{\beta \;(x^{2}+\kappa )}{1+\alpha \kappa +x^{2}}-\delta$$

We consider the system (5.9) along the optimal trajectory where x(t) and y(t) represent the state variables along the optimal path. Looking at the above expansion of the co-efficients, we observe the following:

• $a_{2}(t)>0$
• The sign of $b_{1}(t)$ can be determined based on the sign of the term 1+ακ−κ.
• If $\kappa (t)=\kappa_{\max}$, then $b_{2}(t)>0$ by the hypothesis of the theorem
• $a_{1}(t)$ can either be negative or positive given the values of the state variables and parameters

The characteristic equation of the system (5.9) is given by

(5.10) $$m^{2}+(a_{1}(t)+b_{2}(t))m+(a_{1}(t)b_{2}(t)+a_{2}(t)b_{1}(t))=0$$

We study the qualitative properties of the solution of the system (5.9) based on the properties of the functions $\left(a_{1}(t)+b_{2}(t)\right)$ and $\left(a_{1}(t)b_{2}(t)+a_{2}(t)b_{1}(t)\right)$ used in the characteristic equation (5.10). From Lemma - 1, if we assume that $\frac{-1}{\epsilon }<\lambda (T)<0$, then using the continuity of functions $\left(a_{1}(t)+b_{2}(t)\right)$ and $\left(a_{1}(t)b_{2}(t)+a_{2}(t)b_{1}(t)\right)$, we can imply that there exists a left neighbourhood of *T* in the interval [0,T], say [s,T], such that λ(t)<0 and μ(T)<0 for all t∈[s,T]. As a result, we have $\frac{\partial H}{\partial \kappa }<0$ and consequently $\kappa_{opt}(t)=\kappa_{\max}$. The proof of this theorem would be complete if we can show that s=0.

Now, using the qualitative behaviour of the zero solution of the system (5.9), we will show that the initial values for *λ* and *μ* can be chosen in such a way that λ(t)<0 and μ(T)<0 for all t∈[0,T] thereby proving this theorem. Let us consider two cases based on the sign of the function $b_{1}(t)$.

**Case 1:** 1+ακ−κ≤0

In this case, the function $b_{1}(t)\leq 0$ in the system (5.9). Also, the discriminant of the characteristic equation (5.10) turns out to be positive for all t∈[0,T]. This means that the solution trajectories with initial values chosen such that λ(0)<0 and μ(0)<0 remain in the third quadrant of the *λμ* - space and do not move to any other quadrant thus ensuring that the sign of the switching function does not change throughout in [0,T].

**Case 2:** 1+ακ−κ>0

In this case, $b_{1}(t)>0$ and this alone does not determine the sign of the discriminant of the characteristic equation (5.10). Thus, there is a possibility of the solution of the system (5.9) initiating in the third quadrant of the *λμ* - space to leave that quadrant as time progresses. From the prey-predator expressions at interior equilibrium, we observe that at time t=T, we have x(T)=ϵ and $y(T)>1+\alpha \kappa_{\max}$. This means that the zero solution of the system (5.9) behaves as a saddle when time *t* is closer to the terminal time *T*. Thus, we see that in order to ensure that the solution of the system (5.9) remains in the third quadrant, the initial values must be chosen such that μ(0) is far away from zero on the negative *μ* - axis along with $\frac{-1}{\epsilon }<\lambda <0$ so that when the co-state solution nears negative *λ* - axis as t→T, then the saddle nature of the zero solution prevents it from going out of the third quadrant in the *λμ* - space.

Thus we have $\kappa_{opt}(t)=\kappa_{\max}$ for all t∈[0,T] in both the cases. This proves the theorem. □

### 5.1 Ecological significance of the optimal solutions

The theoretical findings discussed above reveal that the optimal solution obtained can be applied to both biological conservation and pest management. In the former case, the optimal strategy suggests a bang-bang control with a possibility of multiple switches in the trajectory whereas in the latter, there is no switch in the optimal trajectory. The outcomes of the ecological field studies Toft (2005); Wade et al. (2008) are line with the conclusion of Theorem 3. They show that by providing high quality of additional food continuously of large quantities, pest can be managed with minimal amount in the eco-system. However, by providing high quantity of high quality additional food initially, apparent competition arises among predator species Muller and Godfray (1997) which may also lead to eventual prey elimination. On the other hand, by providing high quantity of low quality additional food Putman and Staines (2004), the growth and condition of the predator species would decline, making it detrimental to the survival of the predators. Thus, in order to conserve both species and achieve co-existence in the system, it would be necessary to switch the optimal control in the optimal trajectory.

## 6 Numerical illustrations

**Figure 6:**
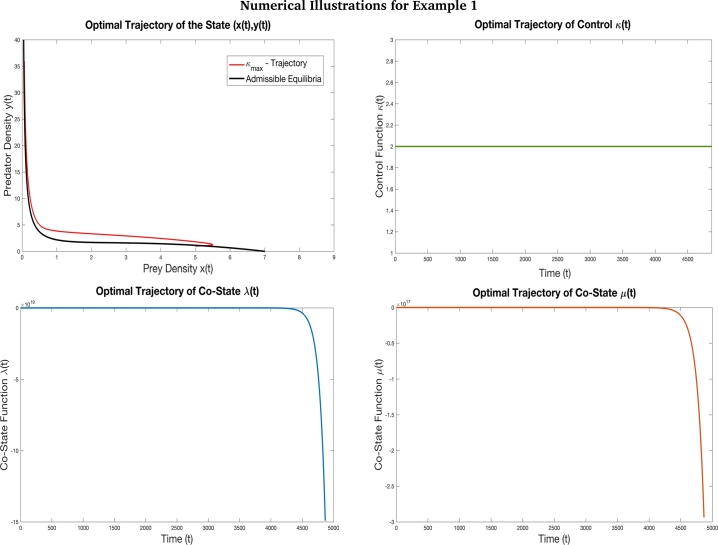
This figure depicts the optimal trajectory of the time optimal control problem (4.1) with the objective to drive the system from the initial state (5,1) to the terminal state (0.05,36) with the parameters values *γ* = 7 *β* = 0.4, *δ* = 0.3, *α* = 0.6, *κ*_min_ = 1, and *κ*_max_ = 2. The initial value chosen for co-state variables is (*λ*(0),*μ*(0))=(−5,−12). Based on the Table 4, for this example we have *P* = −20.90, *Q* = −1.66 and *R* = 1.36 and accordingly *P* < *Q* < *κ*_min_ < *R* < *κ*_max_. Thus, from our earlier analysis, the solution trajectories must tent towards prey elimination. This example shows how the results obtained can be applied in case of pest management. This example illustrates Theorem 3 where the optimal control does not undergo any switch. Also, the co-state variables are negative throughout the optimal trajectory. This example shows that when high quality additional food is provided to the predators in maximum quantity, the prey (pest) can be reduced to a level at which they no longer cause significant damage to the ecosystem. The desired terminal state is reached in *T* = 48.68 units of time.

**Figure 7:**
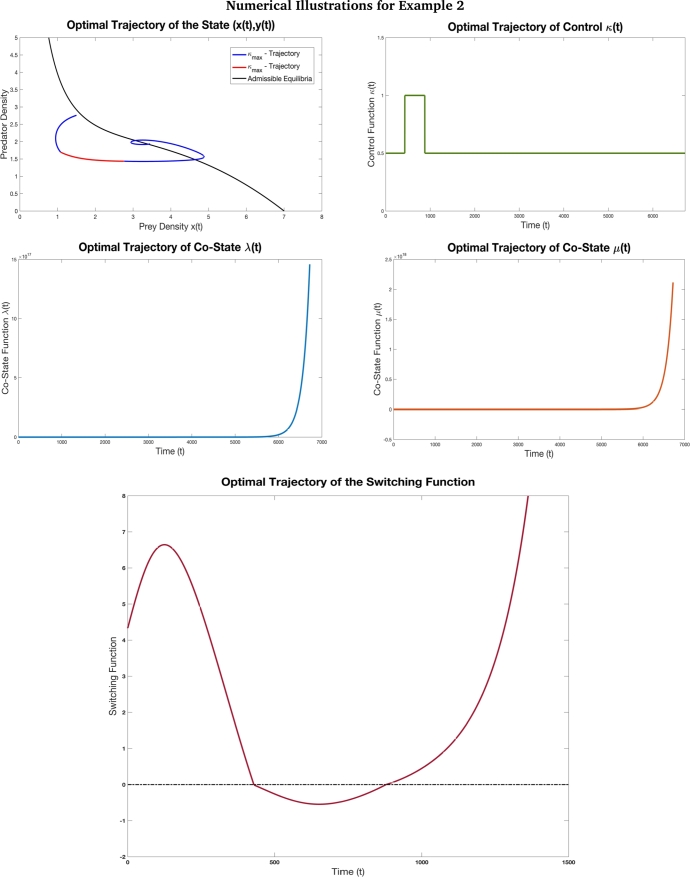
This figure depicts the optimal trajectory and switching function associated with the time optimal control problem (4.1) with the objective to drive the system from the initial state (1.5,2.76) to the terminal state (3.5,1.89) with the parameters values *γ* = 7 *β* = 0.4, *δ* = 0.37, *α* = 0.8, *κ*_min_ = 0.5, and *κ*_max_ = 1. The initial value chosen for co-state variables is (*λ*(0),*μ*(0))=(10,−5). Based on the Table 4, for this example we have *P* = −10.57, *Q* = −1.25 and *R* = 3.55 and accordingly *P* < *Q* < *κ*_*min*_ < *κ*_max_ < *R*. The desired terminal state is reached in *T* = 67.21 units of time. Observe that the optimal control switches twice depending on the switching function. This example depicts the case where multiple switches are involved to bring in co-existence of species.

**Figure 8:**
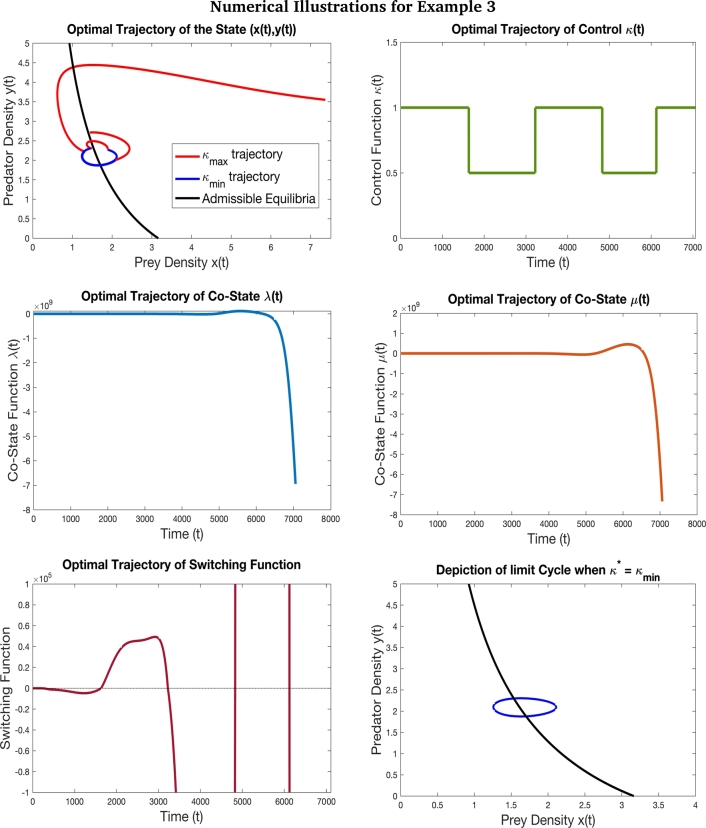
This figure depicts the optimal trajectory and the switching function of the time optimal control problem (4.1) with the objective to drive the system from the initial state (7.37,3.55) to the terminal state (1.39,2.74) with the parameters values *γ* = 8 *β* = 0.4, *δ* = 0.2, *α* = 1.1, *κ*_min_ = 0.5, and *κ*_max_ = 1. The initial value chosen for co-state variables is (*λ*(0),*μ*(0))=(−6,3.5). Based on the Table 4, for this example we have *P* = −87.14, *Q* = 0.369 and *R* = 4.28 and accordingly *P* < *Q* < *κ*_min_ < *κ*_max_ < *R*. Here, we see that at *κ* = *κ*_min_, there is a limit cycle which is formed. The initial state and the terminal state are not in the limit cycle but the optimal trajectory crosses the limit cycle when *κ* = *κ*_min_ (which is depicted in the blue portion of the optimal state trajectory in the figure). This example is a case with four switches in the optimal solution. This case also depicts a situation where the prey density is drastically reduced and not varying the predator density much. We also observe that since additional food is of inferior high quality $1<\alpha <\frac{\beta }{\delta }$, the quantity $\sqrt{\frac{1}{\left(\alpha -1\right)}}>0$ and thus the prey-predator density admissible curve touches the prey axis at $x=\sqrt{\frac{1}{\left(\alpha -1\right)}}$ before *x* = *γ*. Here, the desired terminal state is reached in *T* = 70.58 units of time.

**Figure 9:**
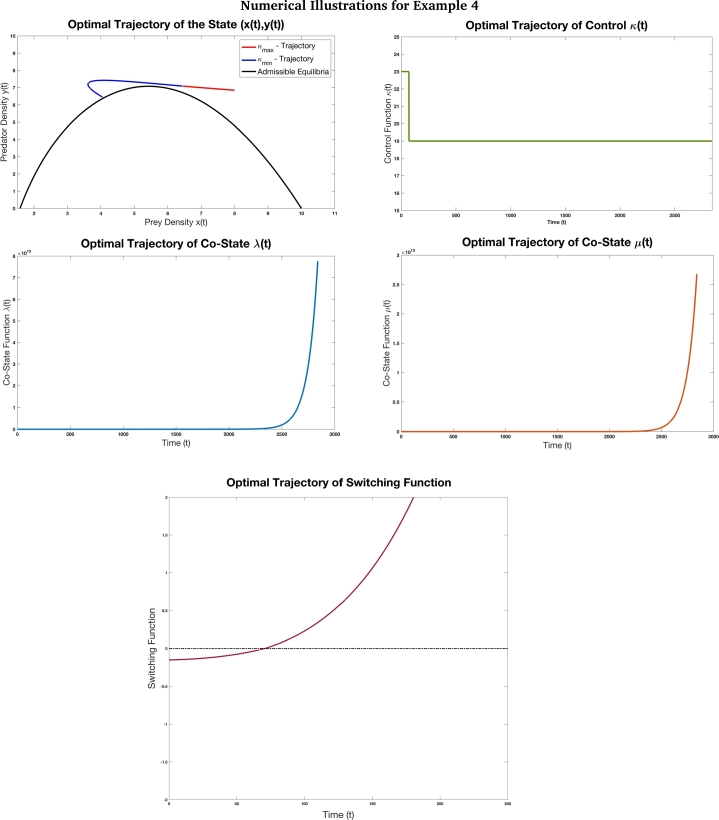
This figure depicts the optimal trajectory and switching function associated with the time optimal control problem (4.1) with the objective to drive the system from the initial state (8,6.85) to the terminal state (4.1,6.4) with the parameters values *γ* = 10 *β* = 0.67, *δ* = 0.54, *ξ* = 1, *α* = 1.4, *κ*_min_ = 19, and *κ*_max_ = 23. The initial value chosen for co-state variables is (*λ*(0),*μ*(0))=(0.05,−13). This is the case where low quality additional is provided to the predators. Based on the Table 4, for this example we have *P* = 144.88, *Q* = 1.44 and *R* = −6.27 and accordingly *R* < *Q* < *κ*_min_ < *κ*_max_ < *P*. Here, the optimal solution undergoes just one switch in its path. The desired terminal state is reached in *T* = 28.39 units of time. In this case, we see that low quality of additional food is provided. This is the reason for the prey-predator admissible curve to be convex with a hump.

In this section, we illustrate the outcomes of the mathematical analysis and results using four examples. These examples are depicted in Figs. 6, 7, 8 and 9. For each of these, the optimal control problem (4.1) and solution were simulated and run on MATLAB software. First we fixed the initial and terminal states of the problem following which we fixed the range of the control parameter $\left[\kappa_{\min},\kappa_{\max}\right]$ using admissible control values. Then, using equation (4.14) and incorporating trial and error method, we obtained the initial values of the co-state variables. The initial control was fixed to one of the extreme values based on the switching function. Using the two state and two co-state variables and a Runge Kutta 4th order routine was used to obtain the solution trajectories. In all cases, the step size was chosen to be h=0.01. Thus, when we discuss the minimum time units obtained for each case, we would scale it down by $10^{-2}$ units. At each time instant during the routine, the switching function was evaluated and switching of the control parameter was based on the sign change of the switching function. Thus, the minimum-time optimal path is illustrated in these examples covering various cases.

These examples re-iterate the importance of supplying additional food by the eco-manager in the right quantity for a fixed quality. In order to achieve co-existence of species leading to biological conservation, the optimal control function could involve multiple switching where as to achieve pest management, it is enough to provide the predators with maximum quantity of high quality additional food.

## 7 Discussion and conclusions

Additional food provision to predators in ecosystems has become an established method in habitat management systems including marine ecosystems to achieve biological conservation and bio-control of species Benelli et al. (2017); Harwood et al. (2004); Putman and Staines (2004); Put et al. (2012); Redpath et al. (2001); Sabelis et al. (2006); Van Baalen et al. (2001); Vandekerkhove and De Clercq (2010); Zhao et al. (2003). Results from the theoretical studies on such systems have identified two key factors that determine the eventual state and stability of the system: the quality and quantity of additional food Srinivasu et al., 2007, Srinivasu et al., 2018; Vamsi et al. (2019). In the study Srinivasu et al. (2018) where additional food system involving type III response is modeled and analysed, outcomes state that with appropriate quality and quantity of additional food, the system can be driven to any state eventually with time. Moreover, by altering these two factors appropriately, not only can the prey and predator species be conserved but also controlled or even eliminated. However, due to the outcomes being asymptotic in nature, practical feasibility and application in real situations becomes difficult.

To overcome this asymptotic dynamics, in the current study, we examined the possibility of driving the type III additional food provided system to the desired terminal state in minimum time with quantity of additional food as the control parameter keeping the quality of additional food *α* a constant. This work is motivated by the experimental studies which showcase the vital role played by the quantity of additional food in maintaining the ecosystems and achieving desired outcomes especially bio-control of pests Beach et al. (2003); Benelli et al. (2017); Ellers et al. (2011); Put et al. (2012); Vandekerkhove and De Clercq (2010); Wade et al. (2008); Winkler et al. (2005); Urbaneja-Bernat et al. (2015). We first analyzed the role of quantity of additional food in the global dynamics of the system. The results showed that to achieve pest management, the quantity of additional food should satisfy the condition $\kappa >\frac{\delta }{\beta -\delta \alpha }$ (Proposition 1), such that the interior equilibrium does not exist. On the other hand, to achieve biological conservation of species, results showed that the system should be driven towards co-existence of species with appropriate quantity of additional food (Proposition 2, Proposition 3, Proposition 4). We identified admissible states which could be the chosen as terminal states that are reached to achieve co-existence. We also saw that not all interior equilibrium points are admissible because there was no well defined quantity that could drive the system to that state.

Once the admissible terminal states were identified, we formulated and studied a time optimal control problem (4.1) to reach the desired terminal state in minimum time. We proved the existence of optimal solution (Theorem 1) using the *Filippov's Existence theorem*. Then we obtained the characteristics of the optimal solution using the *Pontryagin's Maximum Principle*. Using the Hamiltonian minimization condition and the monotonicity property of the Hamiltonian with respect to the quantity parameter *κ*, the optimal control strategy was found to be of bang-bang type with a possibility of multiple switches in the trajectory in case of biological conservation and no switch in case of pest management (Theorem 2, Theorem 3). Since the system (1.5) - (1.6) exhibits contrasting behaviour with respect to quality additional food Srinivasu et al. (2018), we have considered multiple cases of quality as a part of this study and in each case, we fixed the quality parameter *α* as constant. Depending on the position of *α* in comparison with $\frac{\beta }{\delta }$, we classified the additional food into three categories:

(a) Case (i): $\alpha <1<\frac{\beta }{\delta }$ - Superior high quality additional food
(b) Case (ii): $1<\alpha <\frac{\beta }{\delta }$ - Inferior high quality additional food
(c) Case (iii): $1<\frac{\beta }{\delta }<\alpha$ - Low quality additional food

The analysis shows that in case (i), the co-state variables have same the same sign at switch points along the optimal trajectory. In cases (ii) and (iii), the signs of co-state variables at switch times are either same or opposite depending on the position of the quantity $\left(\sqrt{1(\alpha -1)}\right)$ on the prey axis. The prey-predator dependence (equation (3.5)) shows that when additional food is of low quality (case (iii)), the predator density becomes practically undefined ($y^{⁎}<0$) when $x^{⁎}<\sqrt{1/(\alpha -1)}$ and as a result, obtaining pest control strategies is not possible in this case. Thus, pest management is possible only when additional food is of high quality (in cases (i) and (ii)).

The theoretical findings of this work are in line with the ecological field observations. Results from the works Ellers et al. (2011); Putman and Staines (2004) show that when predator is supplied constantly with high quantity of additional food, the effects could be detrimental to the system leading to decrease in survival of either prey or predator depending on the quality of additional food. Thus, in order to achieve biological conservation of species, it is important that there is a switch in the quantity of additional food which would ensure that co-existence of species is maintained. On the other hand, it is also necessary to constantly supply high quantity of high quality additional food in order to control the pest in case of bio-control.

The findings of this study match with the outcomes obtained for studies involving type II functional response Srinivasu and Prasad (2011). However, one unique feature of this study involving type III functional response is that by definition of the response Srinivasu et al. (2018), the admissible predator density (3.5) becomes undefined when prey gets eliminated. Thus, for achieving bio-control, we have considered the strategy of maintaining prey at lowest possible densities (x(T)=ϵ) such that the pests are no longer harmful (instead of considering x(T)=0). This study strategy of maintaining prey at lowest densities is motivated from the following ecological field observations. When *N. tenuis* is used as a natural enemy against whitefiles in tomato crops, it is found that after the pest get eradicated (∼96%), *N. tenuis* start consuming the tomato plant and thereby causing damage to the crops Calvo et al. (2009); Urbaneja-Bernat et al. (2013). Though it is general practice to maintain predator solely with additional food, it could be a good strategy to retain some density of prey rather than depending on supplementary food completely especially when the supplements such as *E. kuehniella* are very costly.

Finally, we have illustrated the theoretical findings by numerically solving four examples covering various cases. These examples validate the results Proposition 5, Theorem 2, Theorem 3. Thus, in conclusion, we have obtained strategies for achieving biological conservation or bio-control in minimum time for additional food system involving type III response. The findings can be useful for eco-manager and experimental scientists who supply additional food to the species.

## Declarations

### Author contribution statement

V.S. Ananth: Conceived and designed the experiments; Performed the experiments; Analyzed and interpreted the data; Contributed reagents, materials, analysis tools or data; Wrote the paper.

D.K.K. Vamsi: Conceived and designed the experiments; Contributed reagents, materials, analysis tools or data.

### Funding statement

This work is partially funded by the Council of Scientific and Industrial Research - Human Resources Department Group (CSIR-HRDG) under the Direct Senior Research Fellowship (Direct - SRF) scheme (File number: 09/0982(11341)/2021-EMR-I).

### Data availability statement

No data was used for the research described in the article.

### Declaration of interests statement

The authors declare no conflict of interest.

### Additional information

Supplementary content related to this article has been published online at https://doi.org/10.1016/j.heliyon.2021.e07699.

No additional information is available for this paper.

## Supplementary material

The following Supplementary material is associated with this article:

Appendices.pdf Appendix. Appendices.pdf
