# Supplementary material for: Influence of quantity of additional food in achieving biological conservation and pest management in minimum-time for prey-predator systems involving Holling type III response
Source: Heliyon. 2021 Aug 3;7(8):e07699. doi: 10.1016/j.heliyon.2021.e07699 (PMC8353493; doi:10.1016/j.heliyon.2021.e07699)
Supplement: Appendices.pdf — Appendix. [file mmc1.pdf]

Appendix

1 Type III Functional Response for Additional Food System

Let N and P denote the prey and predator density respectively and let A denote the biomass of additional food provided. We see that the total time taken by the predator to consume the available food (both the target prey and the additional food) is given by $\Delta T = \Delta T_S + \Delta T_N + \Delta T_A$, where ΔT_S is the time taken by the predator to search the prey, ΔT_N is the time taken by the predator to handle the prey and ΔT_A is the time taken by the predator to handle the additional food provided. we see that the handling time for the prey caught equals the handling time for one prey item times the total number of prey caught and the prey caught is proportional to the search time and the total prey. Hence, number of prey caught is proportional to $\Delta T_S * N (= C * \Delta T_S * N)$, where C is the proportionality constant, which denotes the catchability of the prey and equals $e_1 N$, where, e_1 represents the search time of the predator per unit prey availability. Now, let handling time for one prey item be h_1. Hence, we see that, the total handling time for the prey(ΔT_N) equals h_1 times number of prey caught, which is given by,

$$\Delta T_N = h_1 \Delta T_S N e_1 N \tag{1.1}$$

Now, the handling time for the additional food equals the handling time for one additional food item times the total density of additional food encountered. Also, the additional food encountered is proportional to the search time and the additional food density. Hence, additional food encountered is proportional to $\Delta T_S * A$ $= C_2 * \Delta T_S * A$, where C_2 is the proportionality constant, which denotes the catchability of the additional food and equals $e_2 A$. Here, e_2 represents the search rate of the predator per unit quantity of additional food. Now, let handling time for one additional food item be h_2. Hence, we see that, the total handling time for the additional food(ΔT_A) equals h_2 times additional food encountered, which is given by, $h_2 \Delta T_S$ A $e_2 A$.

The number of prey encountered per unit time is given by, $\frac{\text{Total No. of Prey caught}}{\text{Total Time}}$, which equals, $\frac{\Delta T_S N e_1 N}{\Delta T_S + \Delta T_N + \Delta T_A}$

$$\begin{aligned} &= \frac{e_1 N^2}{1 + e_1 N^2 h_1 + e_2 A^2 h_2} \\ &= \frac{c N^2}{a^2 + N^2 + \frac{h_2 \epsilon_1}{h_1 \epsilon_2} \frac{e_2 \epsilon_2}{e_1 \epsilon_1} A^2} \end{aligned}$$

The quantity of additional food encountered per unit time is given by, $\frac{\text{Total additional food encountered}}{\text{Total Time}}$, which equals,

$$\begin{aligned} &= \frac{\Delta T_S A^2 e_2}{\Delta T_S + \Delta T_S N h_1 e_1 N + \Delta T_S A^2 e_2 h_2} \\ &= \frac{e_2 A^2}{1 + e_1 N^2 h_1 + e_2 A^2 h_2} \\ &= \frac{c \frac{e_2 \epsilon_2}{e_1 \epsilon_1} N^2}{a^2 + N^2 + \frac{h_2 \epsilon_1}{h_1 \epsilon_2} \frac{e_2 \epsilon_2}{e_1 \epsilon_1} A^2} \end{aligned}$$

So, the functional response of the predator towards the prey (the number of prey encountered per unit time), is given by,

$$F(N) = \frac{c N^2}{a^2 + N^2 + \frac{h_2 \epsilon_1}{h_1 \epsilon_2} \frac{e_2 \epsilon_2}{e_1 \epsilon_1} A^2} =$$

and the functional response of the predator towards the additional food (the quantity of additional food encountered per unit time), is given by,

$$G(A) = \frac{c \frac{e_2 \epsilon_2}{e_1 \epsilon_1} N^2}{a^2 + N^2 + \frac{h_2 \epsilon_1}{h_1 \epsilon_2} \frac{e_2 \epsilon_2}{e_1 \epsilon_1} A^2}$$

2 Nature of the Curve Representing Interior Equilibria

The prey-predator relation at the interior equilibrium is given by the equation

$$y^*(\kappa) = \frac{\beta}{\beta - \delta\alpha}\left(1 - \frac{x^*(\kappa)}{\gamma}\right)\left(\frac{1 + (x^*(\kappa))^2(1-\alpha)}{x^*(\kappa)}\right) \tag{2.1}$$

In order to understand the nature of this curve and its changes with respect to the change in the equilibrium prey density, we differentiate $y^*(\kappa)$ in the above equation (2.1) with respect to x^* and we get

$$\frac{dy^*}{dx^*} = \frac{\beta}{\beta - \delta\alpha}\left(\frac{2(\alpha-1)(x^*)^3}{\gamma} - (\alpha - 1) - \frac{1}{(x^*)^2}\right) \tag{2.2}$$

Rearranging the above equation, we get

$$\frac{dy^*}{dx^*} = \frac{\beta}{\beta - \delta\alpha}\left(\frac{2(\alpha-1)(x^*)^3 - \gamma(\alpha-1)(x^*)^2 - \gamma}{\gamma(x^*)^2}\right) \tag{2.3}$$

From (2.1), we also get

$$\kappa = \frac{\delta - (\beta - \delta)(x^*(\kappa))^2}{\beta - \delta\alpha} \tag{2.4}$$

Now, in order to know the emergence of crest and trough on the curve (2.1), we consider the critical points of the curve which we get by solving the equation $\frac{dy^*}{dx^*} = 0$. From the above equation (2.3) we get

$$\frac{dy^*}{dx^*} = 0 \implies 2(\alpha-1)(x^*)^3 - \gamma(\alpha-1)(x^*)^2 - \gamma = 0 \tag{2.5}$$

which is a cubic equation. Since the solution is not easy by manual computation, we have tried obtaining the qualitative nature of roots based on the nature of coefficients. Let $f(x) = 2(\alpha-1)x^3 - \gamma(\alpha-1)x^2 - \gamma$. Using Descarte's Rule of Signs, we see that we can obtain the number of positive or negative roots almost accurately depending on the sign changes of the coefficients. We consider two cases:

1. Case 1 - $\alpha < 1$: When $\alpha < 1$, by considering the coefficients of $f(-x)$, we see that the number of sign-changes in coefficients is 1 and thus the equation (2.5) certainly has one negative real root. The other two could either be real roots or complex conjugates. When the two roots are complex conjugates, then the curve (2.1) is monotonically decreasing (seen frame A in figure - 2) as x^* increases. On the other hand, if the other two roots are real, then both have to be negative or both positive. If both are negative, then the curve (2.1) becomes monotonically decreasing and if both are positive then the curve has a crest and trough (see figure - 1). In this case, from the definition of the curve, we see that as $x^* \to 0$, we have $y^* \to +\infty$ and the curve touches x^* - axis at $x^* = \gamma$. Suppose the remaining roots of equation (2.5) are of opposite signs, then one of the roots is positive which means that the curve changes its nature and starts to increase. This implies that the curve will not be able to touch the x^*-axis at $x^* = \gamma$ which is a contradiction. Thus, with one root of (2.5) being negative, the remaining two roots have to be of the same sign. This scenario is covered by figure - 1 and frame A of figure - 2.

2. Case 2 - $\alpha > 1$: When $\alpha > 1$, by considering the coefficients of $f(x)$, we see that the number of sign-changes in coefficients is 1 and thus the equation (2.5) has one positive real root always. In this case, we have two sub-cases: one with $1 < \alpha < \frac{\beta}{\delta}$ and the other with $1 < \frac{\beta}{\delta} < \alpha$. Since $\alpha > 1$, we see that $\sqrt{\frac{1}{\alpha-1}} > 0$ thus the curve (2.1) meets the prey axis twice in the positive quadrant once at $x = \sqrt{\frac{1}{\alpha-1}}$ and again at $x = \gamma$.Now, let us see how this behaviour is displayed in each sub-case:

   - When $1 < \alpha < \frac{\beta}{\delta}$, we observe that $\beta - \delta\alpha > 0$ and thus as $x^* \to 0$, we have $y^* \to +\infty$ and the curve touches x^* - axis at $x = \sqrt{\frac{1}{\alpha-1}}$ and at $x = \gamma$. Thus, the curve (2.1) is monotonically decreasing and touches $x^* - axis$ at $x = \sqrt{\frac{1}{\alpha-1}}$ first and further decreases till it encounters the positive root of equation (2.5) after which the curve starts to increase and meets x^* - axis at $x = \gamma$. This phenomena can be observed in frames B and C of figure - 2.
   - When $1 < \frac{\beta}{\delta} < \alpha$, we observe that $\beta - \delta\alpha < 0$ and thus, as $x^* \to 0$, we have $y^* \to -\infty$ and here too the curve touches x^* - axis at $x = \sqrt{\frac{1}{\alpha-1}}$ and at $x = \gamma$. Thus, the curve (2.1) is monotonically increasing and touches $x^* - axis$ at $x = \sqrt{\frac{1}{\alpha-1}}$ first and further increases till it encounters the positive root of equation (2.5) after which the curve starts to decrease and meets x^* - axis at $x = \gamma$ thereby creating a concave hump in the first quadrant. This phenomena is depicted in figure - 3.

We see that the remaining two roots of equation (2.5) have to be both negative or complex conjugates owing to the behaviour of the curve (2.1).

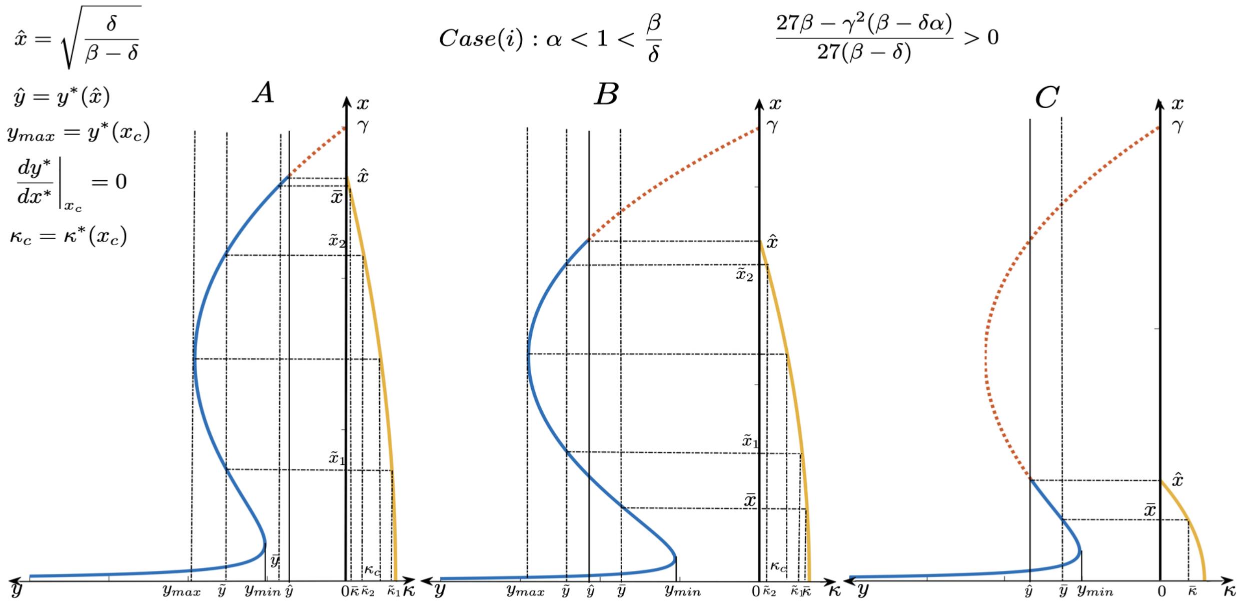

Figure 1: This figure contains two-quadrant graphs which represent the relationship between the admissible equilibria (solid line representing the curve (2.1)) and the quantity of additional food (2.4) when superior high quality additional food is provided and with $\frac{27\beta\gamma^2(\beta-\delta\alpha)}{27\beta-\delta} > 0$. Thus, there exists crest and trough in the curve representing the prey-predator relation leading to possibilities of choosing two different admissible prey densities (and correspondingly two quantities of additional food depending on prey) for a given predator density.

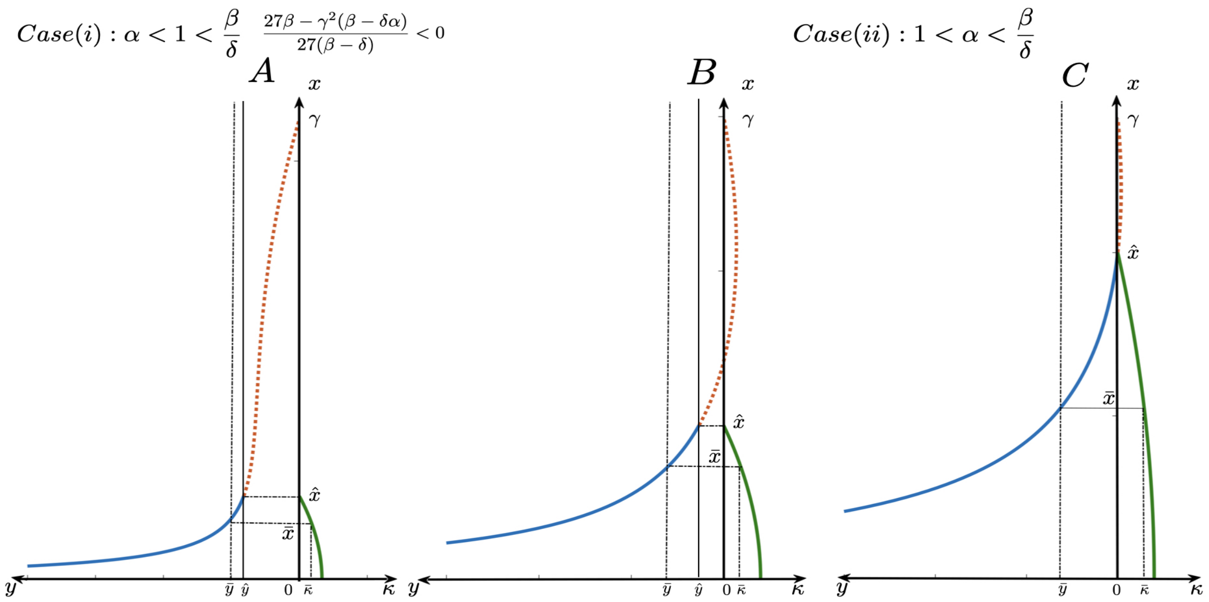

Figure 2: This figure contains two-quadrant graphs which represent the relationship between the admissible equilibria (solid line representing the curve (2.1)) and the quantity of additional food (2.4) where frame A represents the case when superior high quality additional food is provided and with $\frac{27\beta\gamma^2(\beta-\delta\alpha)}{27\beta-\delta} < 0$ where as frames B and C depict the case when inferior high quality additional food is provided to the predators. We note that in both the cases, the curve representing the prey-predator relation is monotonically decreasing with increase in prey density. In these curves, we observe that for a given predator density, there exist unique admissible prey density and corresponding quantity of additional food.

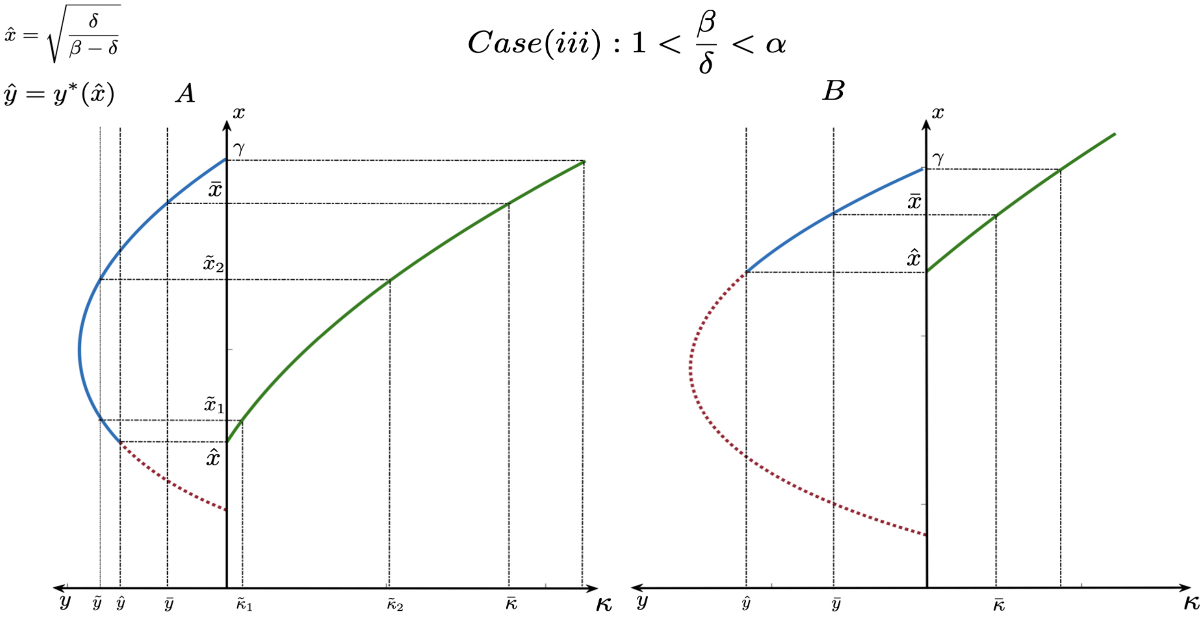

Figure 3: This figure contains two-quadrant graphs which represent the relationship between the admissible equilibria (solid line representing the curve 2.1) and the quantity of additional food (2.4) where predator is provided with low quality additional food. Here too, depending on the position of $\sqrt{\delta/(\beta-\delta)}$, we can get two possibilities of admissible equilibria for a chosen predator density.

Now, we will use the observations made in Srinivasu et al. (2018) regarding the nature of prey isocline to determine under what conditions for Case 1 above ($\alpha < 1$) do we get a monotonically decreasing curve and under what conditions we get a crest and trough. Results from Srinivasu et al. (2018) reveal that for prey isocline curve to have a crest and trough, the following condition must hold:

$$\gamma > 3\sqrt{3(1+\alpha\kappa)} \tag{2.6}$$

We also have from the predator isocline that

$$(x^*(\kappa))^2 = \frac{\delta - (\beta - \delta\alpha)\kappa}{\beta - \delta}$$

Substituting for κ from the above equation, we get the inequality

$$(x^*)^2 = \frac{27\beta - \gamma^2(\beta - \delta\alpha)}{27(\beta - \delta)} \tag{2.7}$$

which implies that for instability to occur in the system by emergence of a crest and trough in the prey isocline, we must have

$$x^* < -\sqrt{\frac{27\beta - \gamma^2(\beta - \delta\alpha)}{27(\beta - \delta)}},\ x^* > \sqrt{\frac{27\beta - \gamma^2(\beta - \delta\alpha)}{27(\beta - \delta)}} \tag{2.8}$$

Since we are not interested in the negative population, the first inequality is not relevant. From the second inequality, we can infer that whenever $\sqrt{\frac{27\beta-\gamma^2(\beta-\delta\alpha)}{27(\beta-\delta)}} > 0$, there is emergence of a crest and trough in the curve (2.1).

Thus, in conclusion, we summarize the above discussion that when $\alpha < 1$, the curve (2.1) has a crest and trough if $\sqrt{\frac{27\beta-\gamma^2(\beta-\delta\alpha)}{27(\beta-\delta)}} > 0$ (refer figure - 1) and monotonically decreasing otherwise (refer frame A of figure - 2). On the other hand, when $\alpha > 1$, then depending on whether $\beta - \delta\alpha > 0$ or $\beta - \delta\alpha < 0$, we get a monotonically decreasing curve (see frames B and C of figure - 2) or a concave shaped curve with a hump in the positive quadrant (refer figure - 3).

,

3 General form of Mayer Problem of Optimal Control

We will present here the general form of *Mayer Problem of Optimal Control* as stated in Cesari (2012). Let A be a subset of the $t\mathbf{x}$ - space $\mathbb{R}^{1+n}$, let U be a given subset of the $\mathbf{u}$ - space $\mathbb{R}^m$. Let $\mathbf{f}(t, \mathbf{x}, \mathbf{u}) = (f_1, f_2, ..., f_n)$ be a given function on $A \times U$. For every $(t, \mathbf{x}) \in A$, let $Q(t, \mathbf{x}) = \mathbf{f}(t, \mathbf{x}, U) \subset \mathbb{R}^n$ be the set of all $z = (z_1, z_2, ..., z_n)$ with $z_i = f_i(t, \mathbf{x}, \mathbf{u}),\ i = 1, 2, ..., n$ for some $\mathbf{u} \in U$. Let B be a given subset of $t_1\mathbf{x}_1t_2\mathbf{x}_2$ - space $\mathbb{R}^{2n+2}$. The Mayer problem of optimal control is to find the optimal solution, usually to minimize the functional

$$I[\mathbf{x}, \mathbf{u}] = g(t_1, \mathbf{x}(t_1)t_2\mathbf{x}(t_2)) \tag{3.1}$$

for pairs of functions $\mathbf{x}(t) = (x_1, x_2, ..., x_n)$, $\mathbf{u}(t) = (u_1, u_2, ..., u_m)$, $t_1 \leq t \leq t_2$, $\mathbf{x}$ absolutely continuous, $\mathbf{u}$ measurable satisfying

$$\frac{d\mathbf{x}}{dt} = \mathbf{f}(t, \mathbf{x}(t), \mathbf{u}(t)),\ t_1 \leq t \leq t_2 \tag{3.2}$$

boundary conditions

$$e[\mathbf{x}] = (t_1, \mathbf{x}(t_1)t_2\mathbf{x}(t_2)) \in B \tag{3.3}$$

and constraints

$$(t, \mathbf{x}(t)) \in A,\ t_1 \leq t \leq t_2, \tag{3.4}$$

$$\mathbf{u}(t) \in U\ t_1 \leq t \leq t_2. \tag{3.5}$$

in the class Ω of all admissible pairs $(\mathbf{x}, \mathbf{u})$. By an admissible pair for the problem (3.1) - (3.5) we mean a pair $(\mathbf{x}(t), \mathbf{u}(t))$, $t_1 \leq t \leq t_2$, x absolutely continuous, $\mathbf{u}$ measurable, satisfying all the requirements (3.1) - (3.5). Here, $\mathbf{x}$ and $\mathbf{u}$ are also called an admissible trajectory and admissible control respectively.

We will now state the *Filippov's Existence Theorem* which is used to prove the existence of an optimal solution to the optimal control problem (3.1) - (3.5).

Theorem 1. *(The Filippov's Existence Theorem for Mayer Problem of Optimal Control) If A and U are compact, B closed, $\boldsymbol{f}$ is continuous on $A \times U$, g is continuous on B, $\Omega \neq \Phi$, and for every $(t, \boldsymbol{x}) \in A$ the set $Q(t, \boldsymbol{x}) = \boldsymbol{f}(t, \boldsymbol{x}, U) \subset \mathbb{R}^n$ is convex, then the objective functional $I[\boldsymbol{x}, \boldsymbol{u}]$ has an absolute minimum in Ω.*

4 Positivity and Boundedness of Additional food provided system

We will establish the positivity and boundedness of the solutions of the additional food provided system:

$$\frac{dx}{dt} = x\left(1-\frac{x}{\gamma}\right)-\left(\frac{x^2y}{1+\alpha\kappa+x^2}\right) \tag{4.1}$$

$$\frac{dy}{dt} = \beta\left(\frac{x^2+\kappa}{1+\alpha\kappa+x^2}\right)y-\delta y \tag{4.2}$$

Theorem 2. *Let* $\boldsymbol{B}=\left\{(x,y)\in\mathbb{R}^2_+ : 0\le x\le\gamma,\ 0\le x+\frac{1}{\beta}y\le\frac{M}{\kappa},\ \kappa>0\right\}$, *with* $M=\frac{\gamma(1+\kappa)^2}{4}$. *If the parameters of the additional food provided system satisfy* $\delta-(\beta-\delta\alpha)\,\xi^2>0$, *then the solutions of the system (4.1) - (4.2) with initial condition* $(x(0),y(0))\in\boldsymbol{B}$ *are bounded in* $\boldsymbol{B}$.

Proof. From the equation (4.1), we see that

$$\frac{dx}{dt}=x\left(1-\frac{x}{\gamma}\right)-\left(\frac{x^2y}{1+x^2+\alpha\xi^2}\right)\le x\left(1-\frac{x}{\gamma}\right)$$

which implies that

$$x\le\frac{\gamma}{1-Ce^{-t}}$$

where C is a constant $\left(\ge 1-\frac{\gamma}{x(0)}\right)$. Thus, we observe that as t becomes large enough, we get $x\le\gamma$ Srinivasu et al. (2018).

Now, let us define $\omega=x+\frac{1}{\beta}y$. Now for any $\kappa>0$, consider the ordinary differential equation

$$\begin{aligned}
\frac{d\omega}{dt}+\kappa\omega &= x\left(1-\frac{x}{\gamma}\right)-\frac{x^2y}{1+\alpha\xi^2+x^2}+\frac{(x^2+\xi^2)y}{1+\alpha\xi^2+x^2}-\frac{\delta}{\beta}y+\kappa x+\frac{\kappa}{\beta}y\\
&= x\left(1-\frac{x}{\gamma}\right)+\frac{\xi^2y}{1+\alpha\xi^2+x^2}+\kappa x+\frac{\kappa-\delta}{\beta}y\\
&= x\left(1-\frac{x}{\gamma}+\kappa\right)+y\left[\frac{\xi^2}{1+\alpha\xi^2+x^2}+\frac{\kappa-\delta}{\beta}\right]
\end{aligned}$$

Since $x\left(1-\frac{x}{\gamma}+\kappa\right)\le\frac{\gamma(1+\kappa)^2}{4}$, we get

$$\begin{aligned}
\frac{d\omega}{dt}+\kappa\omega &\le \frac{\gamma(1+\kappa)^2}{4}+y\left[\frac{\xi^2}{1+\alpha\xi^2+x^2}+\frac{\kappa-\delta}{\beta}\right]\\
&\le \frac{\gamma(1+\kappa)^2}{4}+y\left[\frac{\xi^2}{1+\alpha\xi^2}+\frac{\kappa-\delta}{\beta}\right]\ \text{(by positivity of } x^2)
\end{aligned}$$

Now by choosing κ sufficiently small ($\kappa\ll\delta$) and using condition $\delta-(\beta-\delta\alpha)\,\xi^2>0$ from the hypothesis, we get the relation

$$\frac{d\omega}{dt}+\kappa\omega\le M \tag{4.3}$$

where $M=\frac{\gamma(1+\kappa)^2}{4}$. Now, from the above inequality (4.3) and using the Comparison theorem **?** for the solutions of differential equations $\frac{d\omega}{dt}+\kappa\omega=0$ and $\frac{d\omega}{dt}+\kappa\omega=M$, we get the relation

$$0<\omega(t)<\frac{M}{\kappa}(1-\exp(-\kappa t))+\omega(0)\exp(-\kappa t) \tag{4.4}$$

Now, from (4.4) we see that as t becomes large enough, we get $0\le\omega(t)\le\frac{M}{\kappa}$ Srinivasu et al. (2018); Bandyopadhyay and Chakrabarti (2003). This condition ensures that the additional food provided system is dissipative with respect to the asymptotic bound $\frac{M}{\kappa}$. This bound for $\omega(t)$ guarantees the existence of the compact neighbourhood $\mathbf{B}\subset\mathbb{R}^2_+$. Hence, the solutions of the additional food provided system (4.1) - (4.2) with initial conditions $(x_0,y_0)\in\mathbf{B}$ will be within the set $\mathbf{B}$ if the parameters satisfy the condition $\delta-(\beta-\delta\alpha)\,\xi^2>0$. □

References

Malay Bandyopadhyay and CG Chakrabarti. Deterministic and stochastic analysis of a nonlinear prey-predator system. *Journal of Biological Systems*, 11(02):161–172, 2003.

Lamberto Cesari. *Optimization theory and applications: problems with ordinary differential equations*, volume 17. Springer Science & Business Media, 2012.

PDN Srinivasu, DKK Vamsi, and VS Ananth. Additional food supplements as a tool for biological conservation of predator-prey systems involving type iii functional response: A qualitative and quantitative investigation. *Journal of theoretical biology*, 455:303–318, 2018.
